# Genetic manipulation of *Leishmania donovani* threonyl tRNA synthetase facilitates its exploration as a potential therapeutic target

**DOI:** 10.1371/journal.pntd.0006575

**Published:** 2018-06-13

**Authors:** Sanya Chadha, Ramachandran Vijayan, Sakshi Gupta, Manoj Munde, Samudrala Gourinath, Rentala Madhubala

**Affiliations:** 1 School of Life Sciences, Jawaharlal Nehru University, New Delhi, India; 2 School of Physical Sciences, Jawaharlal Nehru University, New Delhi, India; McGill university, CANADA

## Abstract

**Background:**

Aminoacyl tRNA synthetases are central enzymes required for protein synthesis. These enzymes are the known drug targets in bacteria and fungi. Here, we for the first time report the functional characterization of threonyl tRNA synthetase (*Ld*ThrRS) of *Leishmania donovani*, a protozoan parasite, the primary causative agent of visceral leishmaniasis.

**Methodology:**

Recombinant *Ld*ThrRS (r*Ld*ThrRS) was expressed in *E*. *coli* and purified. The kinetic parameters for r*Ld*ThrRS were determined. The subcellular localization of *Ld*ThrRS was done by immunofluorescence analysis. Heterozygous mutants of *LdThrRS* were generated in *Leishmania* promastigotes. These genetically manipulated parasites were checked for their proliferation, virulence, aminoacylation activity and sensitivity to the known ThrRS inhibitor, borrelidin. An *in silico* generated structural model of *L*. *donovani* ThrRS was compared to that of human.

**Conclusions:**

Recombinant *Ld*ThrRS displayed aminoacylation activity, and the protein is possibly localized to both the cytosol and mitochondria. The comparison of the 3D-model of *Ld*ThrRS to human ThrRS displayed considerable similarity. Heterozygous parasites showed restrictive growth phenotype and had attenuated infectivity. These heterozygous parasites were more susceptible to inhibition by borrelidin. Several attempts to obtain ThrRS homozygous null mutants were not successful, indicating its essentiality for the *Leishmania* parasite. Borrelidin showed a strong affinity for *Ld*ThrRS (K_D_: 0.04 μM) and was effective in inhibiting the aminoacylation activity of the r*Ld*ThrRS (IC_50_: 0.06 μM). Borrelidin inhibited the promastigotes (IC_50_: 21 μM) stage of parasites. Our data shows that *Ld*ThrRS is essential for *L*. *donovani* survival and is likely to bind with small drug-like molecules with strong affinity, thus making it a potential target for drug discovery efforts.

## Author summary

Aminoacyl tRNA synthetases (aaRSs) are ubiquitous enzymes required for protein translation. They play a vital role in helping an organism's survival. Therefore, they have been suggested as favourable targets for the development of antileishmanial drugs. *Leishmania*, a protozoan parasite that causes leishmaniasis is known to encode 26 aaRSs. In the present study, we have worked on the functional characterization of *L*. *donovani* threonyl tRNA synthetase (*Ld*ThrRS) protein. We report that the *L*. *donovani* encodes a functional copy of ThrRS. The protein is localized in the cytosol and possibly also in mitochondria. The *Ld*ThrRS seems to be an essential gene for the parasite since null mutants did not survive. The deletion of one allele of the gene caused reduced growth and attenuated virulence in the heterozygous parasites. These parasites showed increased sensitivity to the known ThrRS inhibitor, borrelidin. Furthermore, borrelidin was found to inhibit the aminoacylation activity of *Ld*ThrRS thus, indicating that parasitic ThrRS can be exploited as a drug target for antileishmanial chemotherapy.

## Introduction

Visceral Leishmaniasis also known as kala-azar, is caused by *Leishmania donovani* [1,2], a protozoan parasite transmitted by the bite of female sand fly [3]. VL is endemic in more than 62 countries with 200 million people at risk of infection [4,5]. VL is the most severe form of leishmaniasis and is lethal if untreated. Due to lack of an effective vaccine against VL, chemotherapy constitutes the primary approach to treat the disease [2]. Increased toxicity and development of resistance against existing repertoire of antileishmanial drugs [6,7], leads to an urgent need for identifying new drugs and drug targets.

The protein translation machinery has been recommended as a target in a wide range of microbes for commercial antibiotics. Most antibiotics interact with microbial ribosomes to target translation apparatus [8]. However, other molecules within the translation process can act as drug targets. Aminoacyl tRNA synthetase family is one such drug target that is employed for existing and future antimicrobial therapeutics. The aaRSs are essential enzymes required during protein translation. These family of enzymes catalyze the esterification of specific amino acid to their corresponding tRNA also known as charged tRNA; this charged tRNA are substrates for protein translation [9]. The aaRSs are not only responsible for providing raw materials for protein translation but also ensure fidelity of translation. The aaRSs are divided into two classes (class I and class II). These classes have been distinguished on the basis of different structural folds and by the site of catalysis [10]. Compounds that inhibit aaRSs have been reported in bacteria as well as in fungus. Mupirocin, an antibacterial drug that acts through inhibition of isoleucyl tRNA synthetase, is currently in clinical use against *Staphylococcus aureus* [8]. These enzymes have been a focus of recent research against eukaryotic parasites. Recently, cladosporin has been shown to target lysyl tRNA synthetase of *Plasmodium falciparum* [11], *L*. *donovani* [12], *Loa loa*, and *Schistosoma mansoni* [13], thus providing a potential lead for anti-parasitic drug discovery. The aaRSs span different amino acid specificities and enzyme classes. Thus, these enzymes appear to be appealing targets for the discovery of new anti-parasitic agents.

Threonyl tRNA synthetase (ThrRS) couples L-threonine to cognate tRNAs and belongs to the class II aaRSs. In *Escherichia coli* ThrRS (*Ec*ThrRS), the dimeric core is formed which is surrounded by the catalytic and anticodon-binding domain. Two additional domains project outside the core at the N-terminal side of each monomer, forming the editing domains of the dimer, which also functions as a binding interface to the tRNA acceptor stem [14]. In comparison to other synthetases, ThrRS charges only a small number of non-cognate amino acids, such as β-hydroxynorvaline and serine [15].

Our earlier computational and bioinformatic study revealed 26 aaRSs in *Leishmania* [16]. The *Leishmania* genome encodes a single copy of ThrRS [TriTrypDB ID: LdBPK_351420.1] [17], while human encode three copies of ThrRS [16]. Thus, in *Leishmania*, ThrRS must serve a dual role in the cytosol as well as in mitochondria. Therefore, it gives an opportunity to target ThrRS in *Leishmania* which further will interrupt protein synthesis in two translational compartments simultaneously. In order to demonstrate ThrRS as a potential new class of targets in antileishmanial drug discovery, we undertook a comprehensive study to identify the essentiality of ThrRS in *L*. *donovani*. The present study characterizes the enzyme activity of *Leishmania donovani* ThrRS (*Ld*ThrRS). We also studied the genetic validation of *Ld*ThrRS by gene replacement strategy in the promastigote stage of parasites. Heterozygous knock out mutants of *LdThrRS* gene indicates that *LdThrRS* plays an indispensable role in the viability and infectivity of this pathogenic organism. Borrelidin, an 18 membered macrolide polyketide, displayed a strong binding affinity for *Ld*ThrRS and had an inhibitory effect on aminoacylation activity of *Ld*ThrRS. The compound exhibited antileishmanial activity both in the promastigote and amastigote *in vitro*. Additionally, the inhibition profile of genetically modified parasites was evaluated in the presence of borrelidin. Our study provides a platform to explore *Ld*ThrRS as a potential target for future work in a drug discovery program.

## Methods

### Reagents and antibodies

All restriction enzymes and DNA ladders were acquired from New England Biolabs (NEB) (MA, USA). The expression vector pET30a was obtained from Novagen (Germany). Ni^2+^-NTA (nitrilotriacetic acid) agarose beads were obtained from Qiagen (USA). Hygromycin, paromomycin and zeocin were acquired from Sigma Aldrich (Germany). Protein ladders were obtained from Fermentas. Borrelidin was purchased from Abcam (Cambridge, UK). The *in vitro* tRNA transcription kit was obtained from Invitrogen (CA, USA). The anti-*Ld*ThrRS antibody was commercially synthesized in rabbits by Merck (Germany).

### Domain architecture and phylogenetic analysis

ThrRS sequence of *Leishmania* and *Trypanosome* was retrieved from TriTrypDB [17]. The *E*.*coli*, human and yeast ThrRS sequences were obtained from UniProt [18]. PFAM database was used for the domain assignments. Phylogenetic analysis was done by combining a set of sequences from UniProt and NCBI. Multiple sequence alignment of these sequences was done by MUSCLE [19], and the seed sequences obtained were further used for phylogenetic tree generation with the help of web version Fig Tree.

### Strains and culture conditions

*L*. *donovani Bob* (*Ld*Bob strain/MHOM/SD/62/1SCL2D) was used for cell culture. This strain was obtained from Dr Stephen Beverley (Washington University, St. Louis, MO). The wild-type promastigotes were cultured at 22°C in M199 medium (Sigma) supplemented with 100 units/ml penicillin (Sigma), 100 μg/ml streptomycin (Sigma) and 5% heat-inactivated fetal bovine serum (Biowest). The heterozygous parasites (*ThrRS*/*NEO* or *ThrRS*/*HYG*) were maintained in either 300 μg/ml paromomycin or 200 μg/ml hygromycin respectively. The ‘add-back’ line (*ThrRS/NEO[pThrRS*^*+*^*]*) was grown in 800 μg/ml zeocin and 300 μg/ml paromomycin. The ThrRS overexpressing cell line (*WT[pThrRS*^*+*^*]*) was maintained in 800 μg/ml zeocin.

The axenic amastigotes were made according to the standard protocol [20]. Briefly, the late-log promastigotes were adapted to grow in an acidic media (RPMI-1640/25 mM 2-(N-morpholino) ethane sulfonic acid (MES)/pH 5.5), at 26°C. Further, these parasites were grown in RPMI-1640/MES/pH 5.5 at 37 ^o^C with 5% CO_2_.

The J774.A1, mouse monocyte macrophage-like cell line was used for virulence studies. This cell line was obtained from ATCC and was maintained in RPMI1640 medium (Sigma) supplemented with 10% FBS (Biowest), 100 U/ml of penicillin and 100 μg/ml streptomycin (Sigma) at 37°C with 5% CO_2_.

### Protein expression and purification

The *LdThrRS* ORF was amplified from genomic DNA using a forward primer with a flanking BamHI site (5’AAAGGATCCATGTCTGGCAAGAAGAAGGCGGCG 3’) and reverse primer with a flanking NotI site (5’AAGGAAAAAA GCGGCCGCCTAGTACTCCCGGTTATGTGTGTCCGCCAGCT 3’). The amplified gene was cloned into pET30a expression vector and was transformed into *BL21 (DE3)* strain. The recombinant *Ld*ThrRS was induced with 0.3 mM IPTG (isopropyl β-D-thiogalactopyranoside) at 16°C for 16 h. Cells were then harvested by centrifugation at 6000 rpm for 10 min. The pellet was resuspended in the lysis buffer (50 mM Tris, pH 7.5, 150 mM sodium chloride, 5 mM imidazole, 5 mM β-Mercaptoethanol, 0.1 mg/ml lysozyme and 2 mM phenylmethylsulfonyl fluoride). The bacterial cells were lysed by sonication and were further centrifuged at 13000 rpm for 30 min. The clear supernatant was passed through Ni^2+^-NTA agarose beads, and recombinant protein was eluted with rising concentrations of imidazole. The purity of the purified protein was checked by running the eluted fractions on SDS-PAGE.

### Enzyme activity assay

The aminoacylation assays were performed as reported earlier [21]. The template for tRNA^Thr^ was amplified from genomic DNA by using a forward primer with T7 promoter sequence (5’ TAATACGACTCACTATAGGGGCCGCTTAGCACAGTGG 3’) and reverse primer with CCA sequence (5’ TGGAGGCCACTCCGAGAATTGAA 3’). The substrate tRNA^Thr^ was *in vitro* synthesized by using MEGAScript *in-vitro* transcription kit. The synthesized tRNA^Thr^ was refolded at 70°C for 10 min, followed by the addition of 10 mM magnesium chloride and slow cooling at RT. Aminoacylation assays were performed in aminoacylation buffer (30 mM Hepes, pH 7.5, 150 mM NaCl, 30 mM KCl and 40 mM MgCl_2_) with 1 mM DTT, 200 μM ATP, 2 U/mL inorganic pyrophosphatase (PPiase; Sigma), 10 mM L-threonine (Sigma), 8 μM tRNA^Thr^ and 0.4 μM r*Ld*ThrRS. The reaction was performed in clear, flat-bottomed 96 well plates and the reaction mixture was kept at 37°C for 30 min. The free inorganic phosphate was measured by the addition of malachite green at 620 nm. For time course experiment, 10 mM EDTA was added to stop the reaction. For determination of kinetic parameters for L-threonine and tRNA^Thr^, the reaction was performed in varying concentrations of L-threonine or tRNA^Thr^ while other components were kept constant. For *Ld*ThrRS inhibition studies with borrelidin, a reaction mixture of 0.4 μM r*Ld*ThrRS was incubated with different concentrations of borrelidin (1–10,000 nM). The reactions were stopped and measured as described above.

### Western blot analysis

The late-log phase promastigotes and axenic amastigotes were harvested. Cells were then washed with 1X PBS (Phosphate buffered saline) and lysed in lysis buffer (10 mM Tris-Cl, pH 8.0, 5 mM DTT, 10 mM NaCl, 1.5 mM MgCl_2_, 0.1 mM EDTA, 0.5% Triton X-100; 0.3 mM phenylmethylsulfonyl fluoride). Further, the cells were lysed by sonication on ice followed by centrifugation at 13000 rpm. Recombinant *Ld*ThrRS protein and 30 μg of the cell extracts were separated on a 10% SDS-PAGE gel and transferred on to a nitrocellulose membrane. After blocking with 5% bovine serum albumin (BSA), the membrane was incubated with the anti-*Ld*ThrRS antibody (1:1000) for 2 h at room temperature (RT). Subsequently, the membrane was washed with phosphate-buffered saline (1X PBS) containing 0.05% Tween 20 (1X PBS-T) and incubated with horseradish peroxidase (HRP) conjugated anti-rabbit antibody (1:5000). The blot was developed using ECL (enhanced chemiluminescence) kit (Amersham Biosciences) according to the manufacturer's protocol.

### Immunofluorescence analysis

For the subcellular localization of *Ld*ThrRS in *L*. *donovani*, log-phase promastigotes were treated with 1 nM MitoTracker Red CMXRos (Molecular Probes) for 20 min. After washing with 1X PBS, cells were immobilized on poly-L-lysine coated coverslips. The cells were then fixed in paraformaldehyde and permeabilized in 0.5% Triton X-100. Subsequently, cells were blocked in 1% BSA followed by incubation with the anti-*Ld*ThrRS antibody (1:2000) for 2 h at RT. Cells were washed and incubated with Alexa 488 conjugated anti-rabbit IgG antibody (Thermo Fischer Scientific) for 45 min at RT. Cells were then treated with DAPI (Sigma) dye for 15 min. The mounted coverslips were then visualized by confocal laser scanning microscope (Olympus FluoView FV1000 with objective lenses PLAPON 60× O, NA- 1.42).

### Molecular constructs for the replacement of *LdThrRS* alleles

A targeted gene replacement strategy was employed for the disruption of *LdThrRS* gene by using fusion PCR [22]. The flanking regions of *LdThrRS* gene (5’ UTR and 3’ UTR) were PCR amplified from genomic DNA of *Bob* and were fused to drug resistance marker gene hygromycin phosphotransferase gene (*HYG*) or neomycin phosphotransferase gene (*NEO*). The 5’ UTR of *LdThrRS* gene was amplified by using primers A & B_Hyg_ or primers A & B_Neo_ (Table 1). The 3’ UTR of *LdThrRS* gene was amplified by using primers E_Hyg_ & F or primers E_Neo_ & F (Table 1). The *HYG* or *NEO* gene was amplified from vectors pX63-HYG or pX63-NEO respectively, with primers C_Hyg_ & D_Hyg_ or C_Neo_ & D_Neo_ respectively (Table 1). The 5’UTR region was then fused to either of the drug resistance genes by using primers A & D_Hyg_ or A & D_Neo_. This fragment was then finally ligated to 3’UTR using primers A & F, yielding 5’UTR_HYG_3’UTR or 5’UTR_NEO_3’UTR.

**Table 1:** Primers used for the generation of Hyg and Neo specific replacement cassettes.

Primer No.	Primer name	Primer sequence
1.	A	5’CTCCCATCCTTCTCTTGCGGAGTG 3’
2.	B_Hyg_	5’GGTGAGTTCAGGCTTTTTCATGTGGCTGAGGTCAAGTTGCACGC 3’
3.	C_Hyg_	5’GCGTGCAACTTGACCTCAGCCACATGAAAAAGCCTGAACTCACC 3’
4.	D_Hyg_	5’CCTATTTGTCATTAGCAGAGTGGCCTATTCCTTTGCCCTCGGACGAG 3’
5.	E_Hyg_	5’CTCGTCCGAGGGCAAAGGAATAGGCCACTCTGCTAATGACAAATAGG 3’
6.	B_Neo_	5’CAATCCATCTTGTTCAATCATGTGGCTGAGGTCAAGTTGCACGC 3’
7.	C_Neo_	5’GCGTGCAACTTGACCTCAGCCACATGATTGAACAAGATGGATTG 3’
8.	D_Neo_	5’CCTATTTGTCATTAGCAGAGTGGCTCAGAAGAACTCGTCAAGAAG 3’
9.	E_Neo_	5’CTTCTTGACGAGTTCTTCTGAGCCACTCTGCTAATGACAAATAGG 3’
10.	F	5’CCAATACAAGGAACGGAAGAGGTATGC 3’

In order to generate the episomal complementation construct the full-length *Ld*ThrRS coding sequence was amplified by using a forward primer with a flanking XbaI site (5’ CCCTCTAGAATGTCTGGCAAGAAGAAGGCGGCGGA 3’) and reverse primer with a flanking HindIII site (5’ CCCAAGCTTCTAGTACTCCCGGTTATGTGTGTCCGCCAG 3’). This amplified gene was cloned into the pSP72α-zeo-α vector to get pSP72α-zeo-α-*ThrRS* complementation construct. For the confirmation of correct orientation and sequence fidelity, all the fragments and constructs were sequenced.

### Generation of genetically modified parasites

The linear fragments generated by PCR amplification were gel purified, and about 2 μg of each replacement cassette was individually electroporated in log phase promastigotes [23]. The cells were subjected to antibiotic selection depending on the marker gene. Cells resistant to the antibiotic selection were analyzed by PCR using primers shown in Table 2, in order to verify the correct integration of the linear replacement cassette. After confirmation of heterozygous parasites, the second round of transfection was initiated in heterozygous parasites. The genotype of mutants was also confirmed by Southern blot analysis by using standard protocol [24].

**Table 2:** Primers used for the molecular characterization of genetically modified parasites by PCR based analysis.

Primer No.	Primer name	Primer sequence
1.	Primer 1	5’TGTAGAAGTACTCGCCGATAGTGG 3’
2.	Primer 2	5’CATTTGCCACGCTCTGCCGTCAGTAGCA 3’
3.	Primer 3	5’CGCAGCTATTTACCCGCAGGACAT 3’
4.	Primer 4	5’AACGTGCTCATCTTTTCACTGTATAAGACGC 3’
5.	Primer 5	5’ATAGCGTTGGCTACCCGTGATATTGC 3’
6.	Primer 6	5’AACACGGCGGCATCAGAGCAGCCGATTG 3’
7.	Primer 7	5’GCGGACACACATAACCGGGAGTACTAG 3’
8.	Primer 8	5’TCCGCCGCCTTCTTCTTGCCAGACAT 3’
9.	Zeo^FP^	5' ATGGCCAAGTTGACCAGTGCCGTTCC 3'
10.	Zeo^RP^	5' TCAGTCCTGCTCCTCGGCCACGAA 3'

In order to generate the ‘add-back’ line *ThrRS/NEO[pThrRS*^*+*^*]*, the episomal construct (pSP72α-zeo-α-*ThrRS*) was transfected into heterozygous *ThrRS/NEO* parasites. After transfection, these parasites were grown in 800 μg/ml zeocin and 300 μg/ml paromomycin. The overexpressing mutant (*WT[pThrRS*^*+*^*]*) parasites were also generated by transfecting episomal construct into wild-type parasites.

### Growth rate analysis

For growth rate experiments, stationary phase parasites were inoculated at a density of 10^6^ cells/ml in M199 medium with 5% FBS in 25 cm^2^ flasks without respective selection antibiotic at 22°C.The growth of each cell line was analyzed at 24 h intervals for 7 days by using Neubauer hemocytometer. Similar results were consistently obtained after repeating the experiment at least three times with wild-type (WT) and genetically modified parasites.

### Infectivity assay

For the infectivity assay, J774.A1 murine macrophage cell line was seeded at a density of 5 x 10^5^ cells per well on coverslips placed in 6—well flat-bottomed plate. The cells were allowed to adhere to the coverslip. For infection, adherent cells were incubated with stationary phase promastigotes at an MOI of 20:1 for 6 h. After 6 h non-adherent promastigotes were removed by washing with 1X PBS. The cells were subsequently maintained in RPMI1640 containing 10% FBS at 37°C. Intracellular parasite load was visualized by Giemsa staining.

### Cell cycle analysis

The log phase promastigotes were collected at a density of 2 x 10^7^. The cells were washed with 1X PBS and then fixed in ice-cold 30% PBS/70% (v/v) methanol for 1 h at 4°C. After 1 h, fixed cells were centrifuged at 1000 rpm for 10 minutes at 4°C, methanol was removed, and pelleted cells were washed twice with ice-cold 1X PBS and then resuspended in 500 μl 1X PBS containing 100 μg/ml RNase and 20 μg/ml propidium iodide (Sigma-Aldrich, USA). The cells were kept for 45 min at 37°C in the dark. The samples were then examined using BD Biosciences FACS Calibur system using BD Biosciences Cell Quest software. For each sample, data for at least 10,000 events were collected. The percentage of cells in G0/G1, S, or G2/M phases of the cell cycle were determined. The result obtained was analysed by the Modfit Lt. Software.

### Borrelidin inhibition studies

In order to determine the borrelidin susceptibility profile of WT and genetically modified promastigotes, we performed a colorimetric MTT [3-(4, 5- dimethylthiazol-2-yl) -2, 5- diphenyltetrazolium bromide] assay, according to manufacturer’s protocol [25]. The log phase promastigotes were seeded at a density of 2 x 10^5^ cells/well in 96 well plate and were treated with different concentrations of borrelidin in M199 media with 5% FBS. The cells were grown for 72 h at 22°C. Subsequently, 10 μl of MTT (5 mg/ml) was added to each well and was incubated at 37°C for 3 h. The reaction was stopped by addition of 50 μl of stop solution (50% SDS and 50% isopropanol), followed by shaking at 70 rpm at 37°C for 30 min. The absorbance was measured at 570 nm in a microplate reader (SpectraMax M2 from Molecular Devices).

The sensitivity of the WT and genetically modified amastigotes to borrelidin was determined. Infectivity assay was performed using different concentrations of borrelidin. After 72 h treatment of drug, the intracellular parasite load was counted by Giemsa staining of the infected J774.A1 murine macrophages. As a control, we also used miltefosine another known antileishmanial drug, to check its efficacy on the WT and the genetically modified promastigotes.

### Isothermal titration calorimetry experiment (ITC)

ITC experiments were performed at 25°C using MicroCal ITC200 (Malvern Instruments Ltd. UK) [26]. Borrelidin and r*Ld*ThrRS were dissolved in the 1X PBS, pH 7.4 and was degassed gently, immediately before use. The total volume of 40 μl injection syringe was added to a sample cell containing 280 μl of r*Ld*ThrRS. The binding experiment involved 20 x 2 μl injections of borrelidin (typically around 200 μM concentration) into the sample cell containing 20 μM of r*Ld*ThrRS. A constant stirring speed of 300 rpm was maintained to ensure the proper mixing of the injectant. Control experiments were performed under similar conditions by injections of borrelidin into the buffer to correct the heat of ligand dilution. After correction of the heat of dilution, integrated heat effects were analyzed by fitting to one set of site model using Origin software. The data were best fitted to one set of site model, yielding thermodynamic parameters such as dissociation constant (K_D_ = 1/K_A_), the stoichiometry of binding (N) and enthalpy (ΔH). Other thermodynamic parameters (ΔS and ΔG) were calculated using standard equations such as (ΔG = -RTlnK) and (ΔG = ΔH–TΔS).

### Model building and validation

The sequence of *Ld*ThrRS was extracted from UniProt database (UniProt ID: E9BS18) [18]. Protein BLAST (blastp) (https://blast.ncbi.nlm.nih.gov) search was used to find the best template structure for *Ld*ThrRS. The human ThrRS with PDB ID 4TTV found with 60.41% sequence identity having the highest similarity score was used to build the 3D structure using homology modelling server MODELLER [27,28]. The obtained structure was energy minimized with the CHARMM force field [29] with 500 steps of steepest descent and 1000 steps of conjugate gradient till the potential energy showed stability. The model quality was validated with ERRAT version 2.0 (servicesn.mbi.ucla.edu/ERRAT).

### Statistical analysis

Data shown are expressed as mean ± S.D. A value of *p <* 0.05 was accepted as an indication of statistical significance. Results for virulence assay and aminoacylation activity in the cell lysates were entered as column data in GraphPad Prism 5.0 (GraphPad Software, Inc.) and analyzed by Student’s two-tailed *t-test*.

## Results

### Domain architecture and phylogenetic analysis

Multiple sequence alignment of the kinetoplastid ThrRS homologs along with the representative sequences from eukaryotes and prokaryotes were created by using CLUSTAL OMEGA (S1 Fig). The data revealed that *Ld*ThrRS shares high sequence identity with the *Leishmania infantum* (~ 99%) and *Trypanosoma brucei* (~ 71%). *E*. *coli* ThrRS displays ~ 37% identity with *Ld*ThrRS. *Saccharomyces cerevisiae* and *Homo sapiens* share sequence identity of ~ 53% with *Ld*ThrRS. Archaeal sequences (*Archaeoglobus fulgidus* and *Methanocaldococcus jannaschii*) are distant homologs with < 25% identity to *Ld*ThrRS sequence. The conserved residues from different representative sequences of ThrRS have been highlighted in blue (S1 Fig). A comparison of the domain architecture of *Ec*ThrRS, *Ld*ThrRS, *Homo sapiens* ThrRS (*Hs*ThrRS) and *Saccharomyces cerevisiae* ThrRS (*Sc*ThrRS) is shown in S2 Fig. ThrRS has a unique modular structure with four structural domains, and a eukaryotic ThrRS has evolved with specific N-terminal extension. The role of N-terminal extension has been recognized in *S*. *cerevisiae* for enzymatic activity and protein stability [30]. Additionally, ThrRS has a putative editing domain (S1 Fig). This domain has an associated secondary domain (tRNA_SAD). The putative editing domain contains HXXXH motif which is a characteristic feature of metal-dependent hydrolases. Thus, the presence of putative editing domain suggests its role in hydrolysis. ThrRS also comprises TGS domain tethered to the tRNA_SAD domain. This domain is seen in regulatory proteins [31]. Therefore, suggesting that ThrRS may also have some regulatory role.

The phylogenetic analysis of kinetoplastid ThrRS along with bacterial, archaeal, apicomplexan, fungal, plants, insects and mammalian homologs reveals the close relationship of kinetoplastid ThrRS to a fungal enzyme (S3 Fig).

### Characterization of *Ld*ThrRS

**Fig 1:**
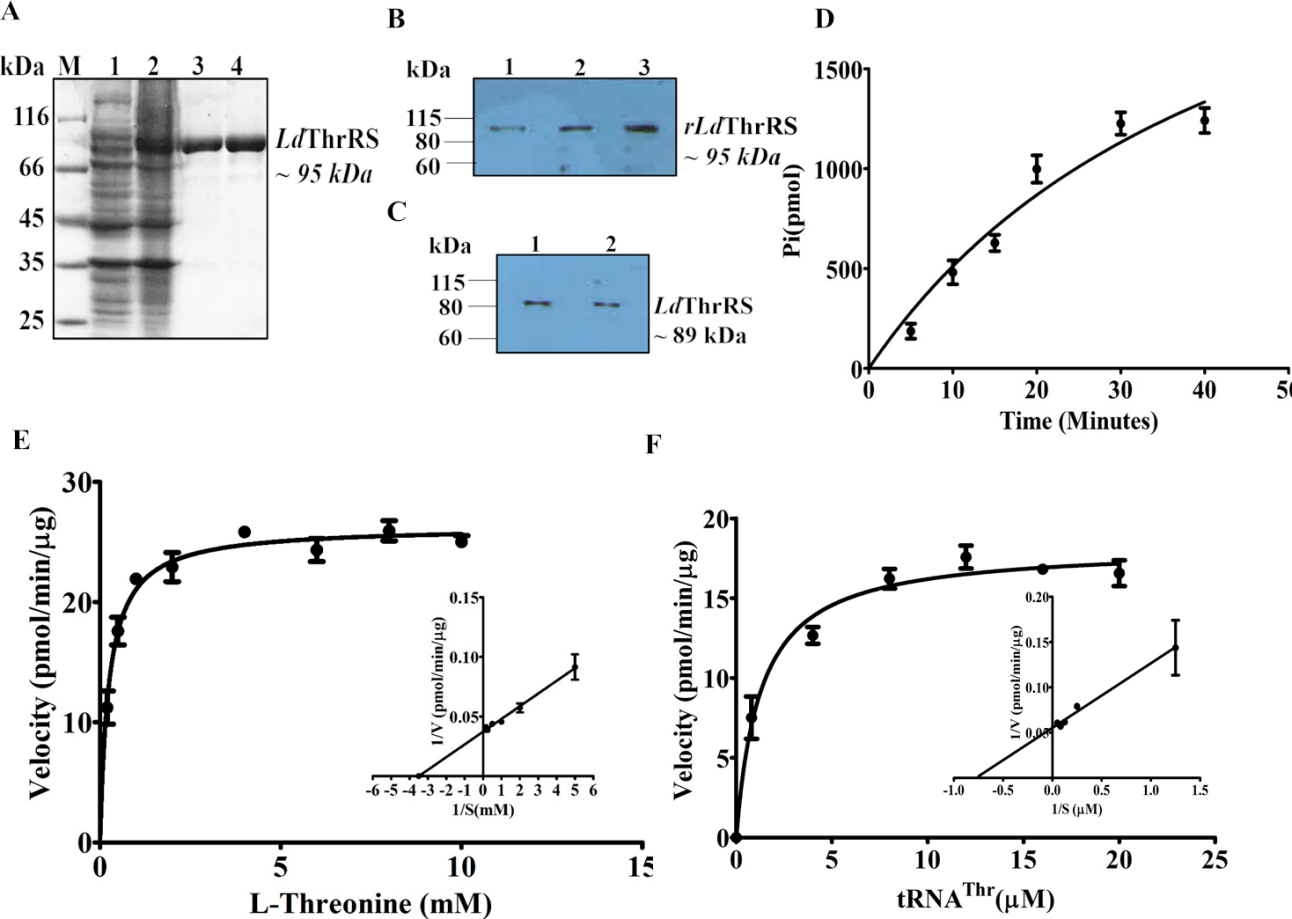
Expression, purification, western blot and enzymatic activity of *Ld*ThrRS. (A) Induction and purification of recombinant *Ld*ThrRS (r*Ld*ThrRS) by Ni^2+-^NTA affinity chromatography. M, molecular weight marker; Lane 1, uninduced cell lysate; Lane 2, induced cell lysate; Lane 3 and 4, eluted fractions with 300 mM imidazole showing purified r*Ld*ThrRS. (B) Western blot analysis of r*Ld*ThrRS with anti-*Ld*ThrRS antibody (1:1000), Lane 1, 0.5 μg of r*Ld*ThrRS; Lane 2, 1 μg of r*Ld*ThrRS; Lane 3, 2 μg of r*Ld*ThrRS. (C) Immunoblot analysis of the cell lysate of 30 μg *Leishmania* with the anti-*Ld*ThrRS antibody (1:1000), Lane 1: promastigote; Lane 2: amastigote. (D) Time-dependent aminoacylation assay of r*Ld*ThrRS. The aminoacylation reactions were performed with L-threonine and tRNA^Thr^ as the substrates. The result shows an average of three different experiments performed in duplicate ± SD. (E) and (F) The aminoacylation kinetics catalyzed by r*Ld*ThrRS as a function of L-threonine (E) and tRNA^Thr^ (F) concentration. The kinetic parameters were calculated by a Michaelis-Menten algorithm within GraphPad Prism 5.0 for utilization of L-threonine and tRNA^Thr^ by the r*Ld*ThrRS enzyme. Results are representative data from three separate experiments and are represented as mean ± S.D.

The recombinant *Ld*ThrRS (r*Ld*ThrRS) was overexpressed, the coding sequence of *Ld*ThrRS was cloned into a pET30a expression vector having an N-terminal His_6_ tag. The cloned gene was transformed into *BL21* (*DE*3) strain and was induced as His_6_-tagged recombinant *Ld*ThrRS with an estimated molecular size of ~ 95 kDa (Fig 1A). The size of r*Ld*ThrRS correlated with the size of *Ld*ThrRS protein (~ 89 kDa) and His_6_ tag (~ 6 kDa). The recombinant His_6_-*Ld*ThrRS was purified by metal affinity chromatography on pre-equilibrated Ni^2+^-NTA agarose resin (Fig 1A). The purified recombinant protein was further confirmed by MALDI-TOF/TOF mass spectroscopy. The r*Ld*ThrRS protein was used to raise polyclonal antibody in rabbits. The western blot analysis recognized a single specific band of the recombinant protein by using anti-*Ld*ThrRS antibody (Fig 1B). The immunoblot analysis was done to confirm the expression of the full-length *Ld*ThrRS protein in cell lysates of promastigotes (Fig 1C, Lane 1) and amastigotes (Fig 1C, Lane 2). The anti-*Ld*ThrRS antibody recognized a single band of ~ 89 kDa in whole cell lysates of *L*. *donovani* (Fig 1C).

The enzymatic activity and kinetic parameters for r*Ld*ThrRS were determined by a spectrophotometric assay. The aminoacylation activity of r*Ld*ThrRS was carried out with r*Ld*ThrRS protein in the presence of pyrophosphatase (PPiase), and the released inorganic phosphate (Pi) was measured with the malachite green. A time course experiment was performed to check the aminoacylation rate over time. We detected an increase in the acylated tRNA^Thr^ formed over time (Fig 1D), which shows that *L*. *donovani* encodes a functional ThrRS enzyme. The kinetic parameters of r*Ld*ThrRS were determined using L-threonine and tRNA^Thr^ as substrates. We performed enzyme kinetics with r*Ld*ThrRS by varying concentrations of one substrate while other components were kept constant. The *K*_*m*_ value of r*Ld*ThrRS for L-threonine was deciphered to be 250 ± 30 μM (Fig 1E). The specific activity of the enzyme under these conditions was 1.7 min^-1^ which is closer to that of *T*. *brucei* (kcat—1.4 min^-1^) [32]. The estimated *K*_*m*_ value of r*Ld*ThrRS, for tRNA^Thr^, was 1.2 ± 0.23 μM (Fig 1F).

### Cell localization of *Ld*ThrRS

**Fig 2:**
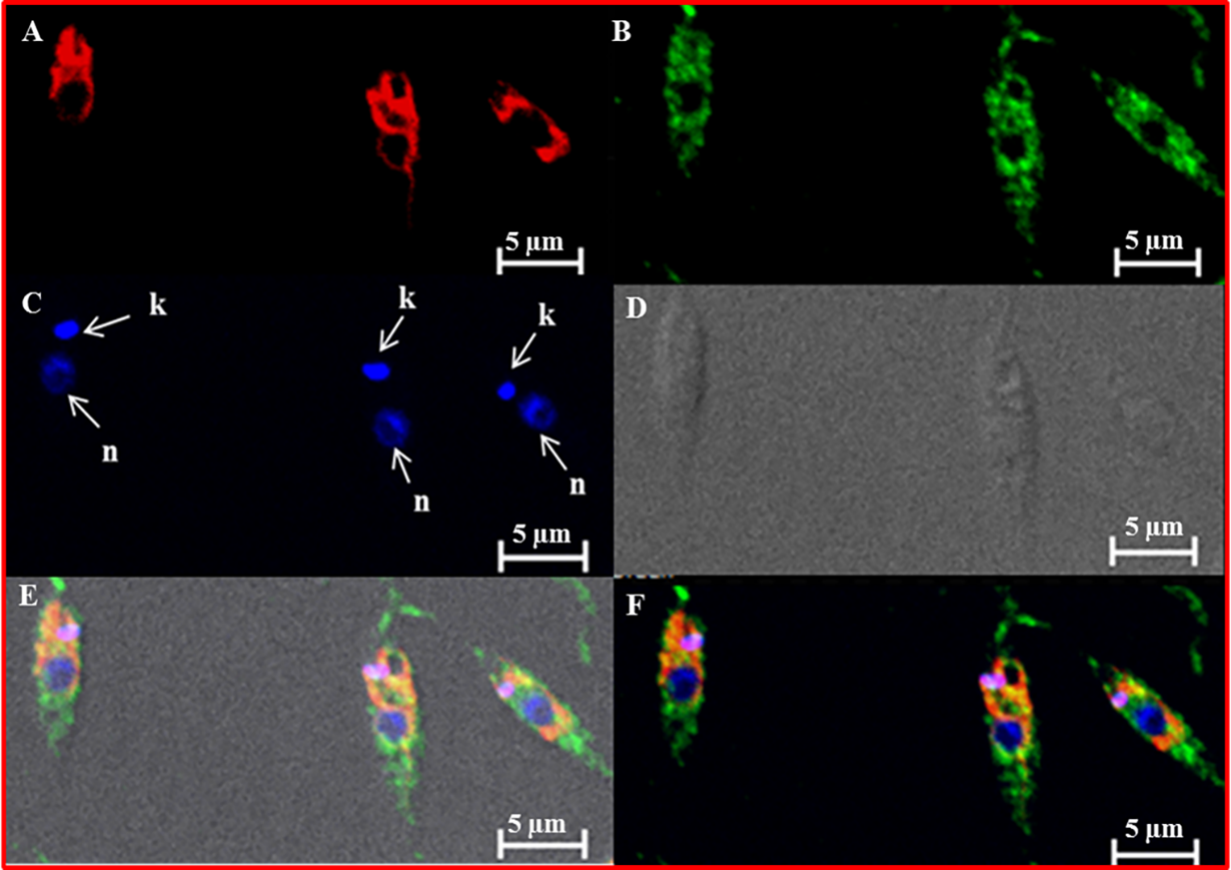
Localization of *Ld*ThrRS in *L*. *donovani*. Immunofluorescence analysis by confocal micrograph of wild-type log phase promastigotes stained with DAPI (C), anti-*Ld*ThrRS antibody detected using Alexa 488 (green)-conjugated secondary antibody (B) and mitotracker red CMXRos (A). (E) and (F) merged micrographs and (D) phase contrast image. ‘k’ and ‘n’ indicate kinetoplastid and nuclear DNA respectively. The scale bar represents 5 μm.

Immunofluorescence analysis was done in order to determine the subcellular localization of ThrRS in promastigote stage of *L*. *donovani*. The *Leishmania* genome encodes a single copy of ThrRS, thus indicating that possibly a dual-purpose ThrRS is encoded by *L*. *donovani* which works both in the cytosol as well as in the mitochondria. The log phase promastigotes were stained with anti-*Ld*ThrRS antibody along with DAPI (nuclei) and mitotracker red (mitochondria) to mark those compartments. A diffused staining of the *Ld*ThrRS was observed, consistent with a cytosolic localization (Fig 2B). However, the merged image also displayed a possible indication for mitochondrial localization as observed by the presence of yellow color along the location of the mitochondria (Fig 2F). Earlier reports have shown cytosolic as well as mitochondrial localization of *Tb*ThrRS in *T*. *brucei* [32].

### Generation of *Ld*ThrRS deletion mutants

In order to determine the importance of *ThrRS* gene in *L*. *donovani*, the *ThrRS* encoding sequence was inactivated by open reading frames encoding for neomycin phosphotransferase (*NEO*) or hygromycin phosphotransferase (*HYG*). Two constructs having neomycin phosphotransferase (*NEO*) or hygromycin phosphotransferase (*HYG*) as selection markers linked to the 5’ and 3’ UTR’s of the *LdThrRS* gene were generated by fusion PCR, as described in the Methods. These constructs were transfected into wild-type (WT) cells leading to the generation of heterozygous parasites (*ThrRS/NEO* or *ThrRS/HYG*) in which one allele of *LdThrRS* gene was replaced with antibiotic resistance gene.

These parasites with one allele of *ThrRS* were re-transfected with the constructs containing the second antibiotic resistance gene to replace the remaining allele of *LdThrRS*. The double knock out parasites did not survive in the culture which suggests essentiality of this gene for parasite survival.

**Fig 3:**
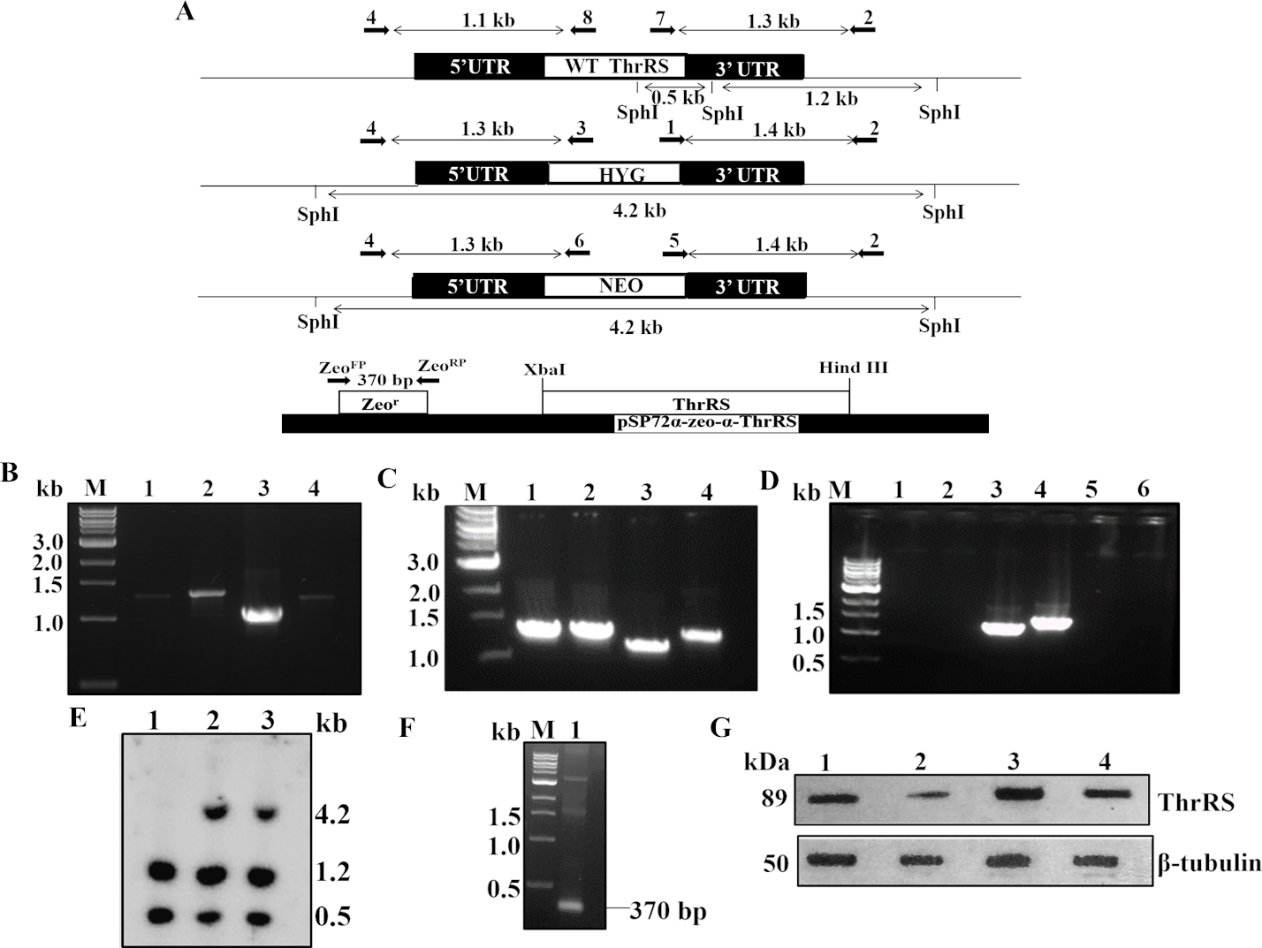
Generation of heterozygous mutants of *LdThrRS*. (A) Map of *LdThrRS* genomic locus and pSP72α-zeo-α-*ThrRS* episomal construct is shown with the position of the primers used for confirmation of WT and mutant parasites by PCR-based analysis along with the expected band sizes. Primer pairs are shown in Table 2. Primer 4 was designed as a forward primer to match the upstream region of *LdThrRS* gene, and primers 8, 3 and 6 were designed internal to *LdThrRS*, *HYG* and *NEO* coding regions, respectively. Primer 2 was designed as a reverse primer to match the downstream region of *LdThrRS* gene and primers 7, 1 and 5 were designed as forward primers, internal to *LdThrRS*, *HYG* and *NEO* coding regions, respectively. Forward primer Zeo^FP^ and reverse primer Zeo^RP^ were designed to match the upstream and downstream region of zeocin resistance gene. Genomic DNA from WT, heterozygous parasites (*ThrRS/NEO* or *ThrRS/HYG*) was used as a template for PCR analysis. (B) The specific fusion of the replacement cassette(s) was checked with *NEO* and gene-specific primers as reported in Table 2 and Fig 3A. Lane 1 (Primers 4 and 6); Lane 2 (Primers 5 and 2); Lane 3 (Primers 4 and 8) and Lane 4 (Primers 7 and 2). (C) The specific fusion of the replacement cassette(s) was checked with *HYG* and gene-specific primers. Lane 1 (Primers 4 and 3); Lane 2 (Primers 1 and 2); Lane 3 (Primers 4 and 8) and Lane 4 (Primers 7 and 2). (D) WT genomic DNA was used as a negative control. The bands corresponding to the WT gene were obtained in Lane 3 and 4. No bands were observed after using *NEO* specific primers, Lane 1 (Primers 4 and 6); Lane 2 (Primers 5 and 2) and *HYG* specific primers, Lane 5 (Primers 4 and 3); Lane 6 (Primers 1 and 2). M indicates the molecular size marker in kb. (E) Southern blot analysis of genomic DNA from wild-type (WT) (Lane 1), *ThrRS/NEO* (Lane 2) and *ThrRS/HYG* (Lane 3) parasites. Genomic DNA from WT, *ThrRS/NEO* and *ThrRS/HYG* parasites were digested with SphI, separated on a 0.6% agarose gel and probed with 3’UTR of *LdThrRS* gene. (F) PCR analysis of rescue mutants (*ThrRS/NEO[pThrRS*^*+*^*]*). The specificity of recombination was checked with *Zeo*^*r*^ specific primers, Lane 1 (Primers Zeo^FP^ and Zeo^RP^). M indicates the DNA molecular size marker. (G) Western blot analysis of WT (Lane 1), heterozygous parasites (*ThrRS/NEO*) (Lane 2), *ThrRS* overexpressors (*WT[pThrRS*^*+*^*]*) (Lane 3) and rescue mutant (*ThrRS/NEO[pThrRS*^*+*^*]*) (Lane 4) parasites. β- tubulin was used as a loading control.

For characterization of these heterozygous parasites, the successful removal of one allele of *ThrRS* gene was confirmed by PCR analysis of genomic DNA of these parasites by using specific primers (Table 2) external to the replacement constructs (Fig 3A). The PCR analysis demonstrated the correct integration of *NEO* and *HYG* replacement cassettes at the *ThrRS* locus in heterozygous (*ThrRS/NEO* or *ThrRS/HYG*), as indicated by the appearance of ~ 1.3 (Fig 3B, Lane 1) and ~ 1.4 kb (Fig 3B, Lane 2) bands in the case of *NEO* cassette and ~ 1.3 (Fig 3C, Lane 1) and ~ 1.4 kb (Fig 3C, Lane 2) bands in the case of *HYG* cassette, along with the ~ 1.1 (Fig 3B and 3C, Lane 3) and ~ 1.3 kb (Fig 3B and 3C, Lane 4) bands corresponding to the WT *LdThrRS* gene. This data confirmed that a single allele of the *LdThrRS* gene had been replaced in heterozygous mutant parasites (*ThrRS/NEO* or *ThrRS/HYG*). The WT genomic DNA was used as a negative control (Fig 3D). The bands corresponding to the WT gene were obtained (Fig 3D, Lane 3 and 4). No bands were observed after using *NEO* (Fig 3D, Lane 1 and 2) and *HYG* (Fig 3D, Lane 5 and 6) specific primers.

Further, Southern blot analysis was done to confirm the genotype of heterozygous parasites (Fig 3E). The expected bands upon digestion with SphI are represented in Fig 3A. After probing with 3’UTR of *LdThrRS* gene, 1.2 kb and 0.5 kb bands of expected size were observed in WT parasites (Fig 3E, Lane 1) and also in heterozygous parasites (Fig 3E, Lane 2 and 3). Heterozygous parasites had an additional band of 4.2 kb corresponding to the *NEO* (Fig 3E, Lane 2) and *HYG* replacement constructs (Fig 3E, Lane 3).

The rescue mutant line (*ThrRS/NEO[pThrRS*^*+*^*]*) were generated by transfecting pSP72α-zeo-α-*ThrRS* construct into heterozygous parasites (*ThrRS/NEO*). These parasites were confirmed for the presence of episome plasmid by PCR (Fig 3F). A band of ~ 370 bp was found upon being amplified by Zeo^FP^ and Zeo^RP^ primers (Fig 3F Lane 1), which corresponds to zeocin resistance gene (*Sh ble* gene), thus confirming the presence of pSP72α-zeo-α-*ThrRS* construct in rescue mutants (*ThrRS/NEO[pThrRS*^*+*^*]*).

We further checked the protein level of ThrRS across genetically modified parasites by Western blot analysis. Comparative densitometric analysis revealed ~ 2-fold decreased expression of ThrRS in heterozygous parasites (*ThrRS/NEO*) (Fig 3G, Lane 2), as compared to the WT cells (Fig 3G, Lane 1). The rescue mutants (*ThrRS/NEO[pThrRS*^*+*^*]*) restored protein level comparable to that of WT parasites (Fig 3G, Lane 4). The overexpressing mutants (*WT[pThrRS*^*+*^*]*) showed ~ 1.5 fold increase in ThrRS level as compared to the WT parasites (Fig 3G, Lane 3).

### Aminoacylation, growth and infectivity studies of genetically modified parasites

**Fig 4:**
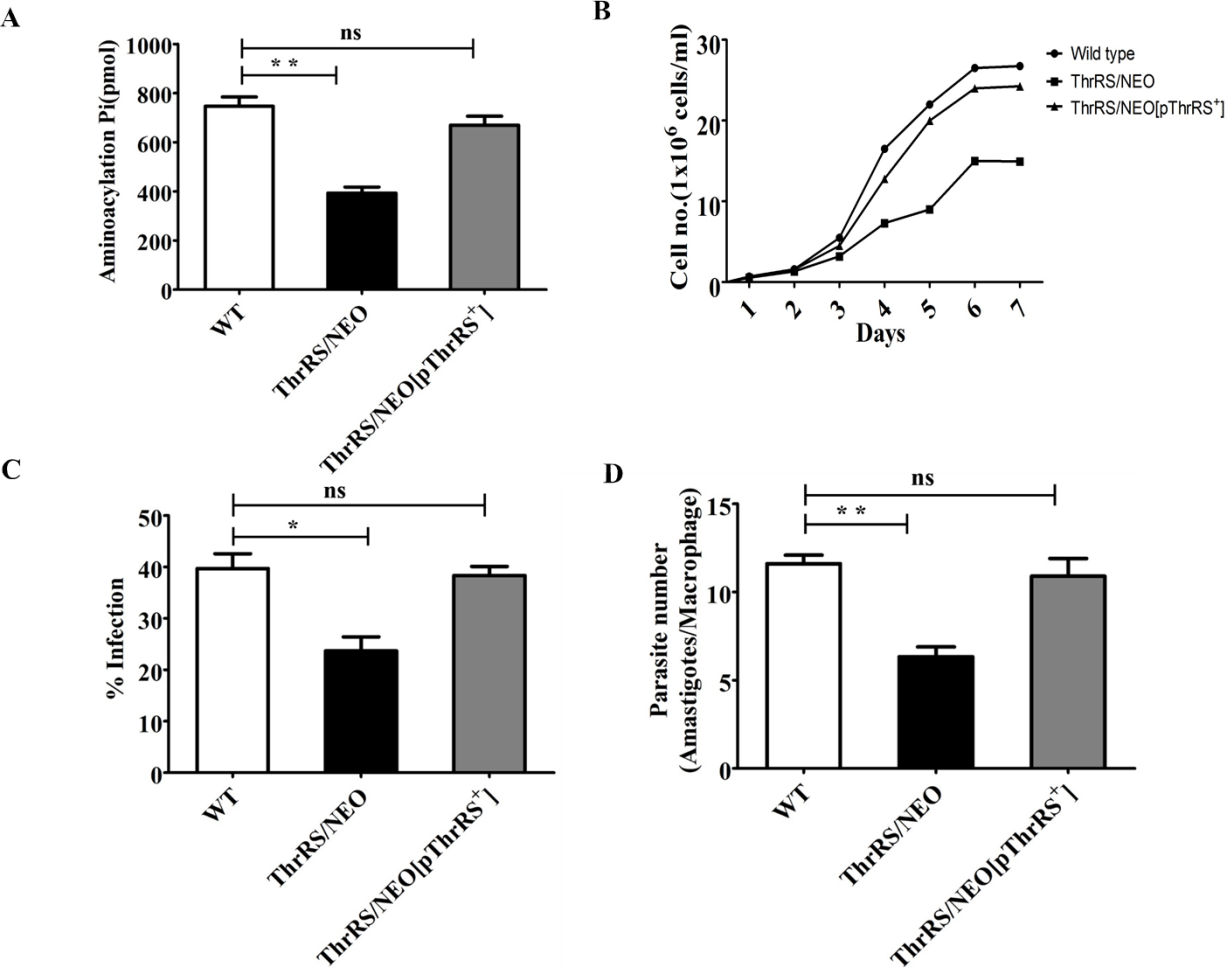
Characterization of genetically modified parasites. (A) Aminoacylation activity of *Ld*ThrRS in the cell lysates of *L*. *donovani* WT, heterozygous *(ThrRS/NEO)* and rescue mutant (*ThrRS/NEO[pThrRS*^*+*^*]*) parasites. (B) Comparison of the growth curve characteristics of WT, heterozygous (*ThrRS/NEO*) and rescue mutant (*ThrRS/NEO[pThrRS*^*+*^*]*) promastigotes in M199 media. The experiment was repeated thrice in triplicate. The data shown here is from one experiment. (C) and (D) Comparison of the infectivity (C) and parasite load (D) of *L*. *donovani* WT, *ThrRS/NEO* and *ThrRS/NEO[pThrRS*^*+*^*]* parasites in J774A.1 murine macrophage cell line. The stationary phase promastigotes were used to infect murine macrophage cell line J774A.1 at an MOI of 20:1. After 48 h of infection, cells were stained, and amastigotes were counted visually. The results signify mean ± S.D with *n* = 3, *P < 0.05, **P < 0.01 statistical difference from the wild-type control.

The disruption of a single allele of ThrRS was assessed to check if it compromised cellular physiology. The aminoacylation activity of heterozygous parasites (*ThrRS*/*NEO*) was 55% of the WT cells (Fig 4A). In the rescue mutants (*ThrRS/NEO[pThrRS*^*+*^*]*), aminoacylation activity of ThrRS was restored and was comparable to that of WT parasites (Fig 4A).

Next, the growth rate of heterozygous parasites and WT cells was examined in order to see if deletion of a single allele of *ThrRS* affects the growth of the parasites. The division rate of heterozygous parasites (*ThrRS*/*NEO*) was significantly reduced than the WT cells resulting in slower growth (Fig 4B). This delayed growth phenotype was rescued in the ‘add-back’ line (*ThrRS/NEO[pThrRS*^*+*^*]*) (Fig 4B).

We further assessed the impact of heterozygous parasites on its ability to infect host cells. Virulence studies were carried out in J774.A1 murine macrophage with the stationary phase parasites at an MOI of 20:1. WT cells were capable of infecting ~ 40% of murine macrophages while heterozygous parasites (*ThrRS*/*NEO*) had reduced infectivity with only ~ 20% macrophages being infected (Fig 4C). The rescue mutants (*ThrRS/NEO[pThrRS*^*+*^*]*) showed infection comparable to that of WT parasites (Fig 4C). Upon comparing the parasite load of WT and heterozygous parasites in the macrophages, it was seen that heterozygous parasites (*ThrRS*/*NEO*) had 50% reduction in the number of parasitemia (amastigotes/macrophage) as compared to WT cells (Fig 4D). In the rescue mutants (*ThrRS/NEO[pThrRS*^*+*^*]*), parasite number was restored to the level of WT cells. Our data suggest that deletion of a single allele of ThrRS parasite has the potency of reducing the ability of amastigotes to multiply *in vitro*. The slow growth phenotype of the heterozygous mutants compared to the wild-type may be partially attributed to the reduced infectivity.

### Flow cytometric and morphological analysis of heterozygous parasites

**Fig 5:**
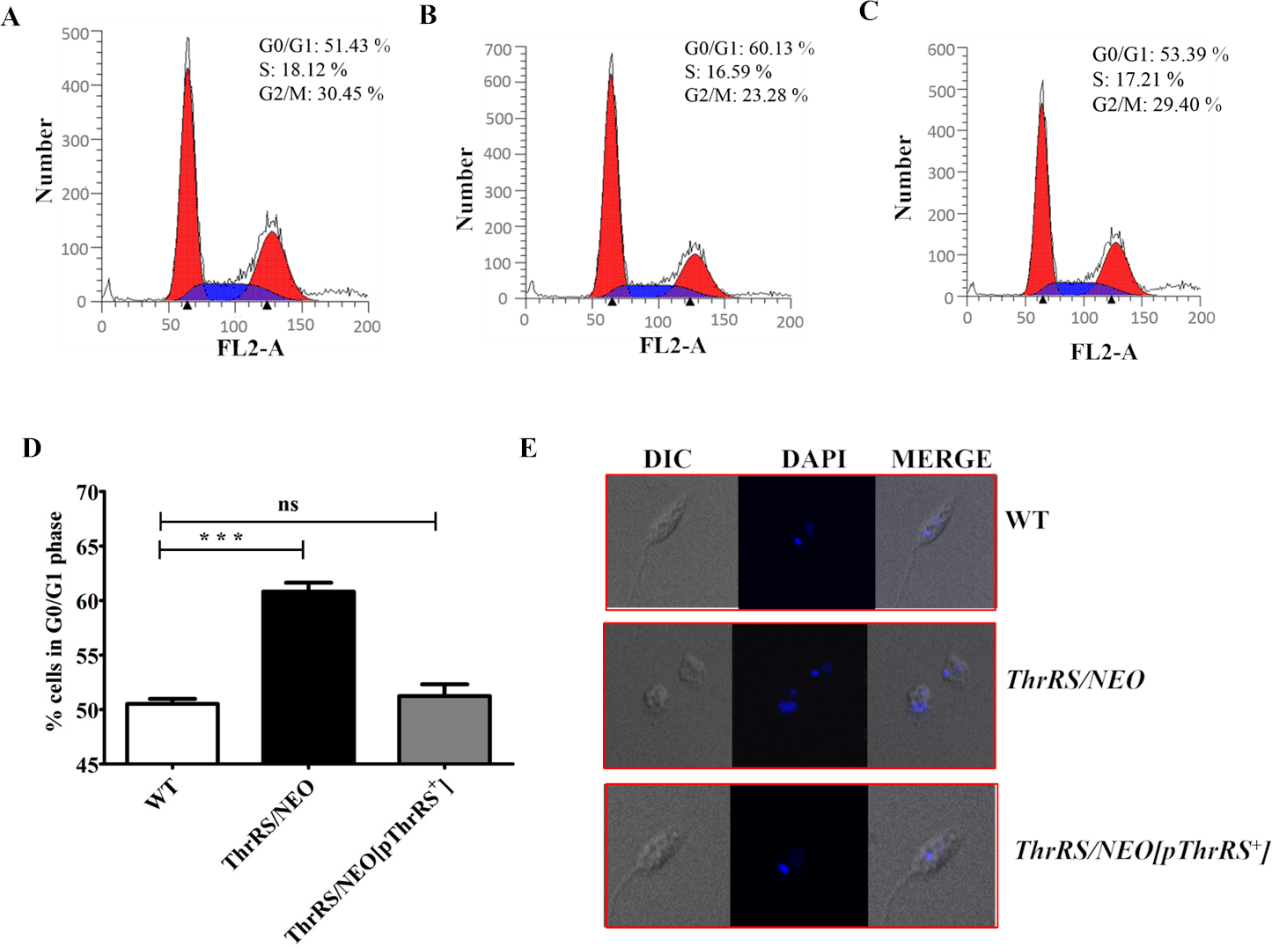
Cell cycle and morphological study of heterozygous parasites. Cell cycle analysis of (A) WT, (B) heterozygous parasites (*ThrRS*/*NEO*) and (C) Rescue mutants (*ThrRS/NEO[pThrRS*^*+*^*]*). PI fluorescence (FL2-Area) is plotted versus cell count. The first peak (red color) indicates cells in G0/G1 phase, S phase is indicated in blue color, and third peak (red color) represents cells in G2/M phase. (D) A bar graph is representing the percentage of cells in the G0/G1 phase of WT, *ThrRS*/*NEO* and *ThrRS/NEO[pThrRS*^*+*^*]* parasites. The results represent mean ± S.D with *n* = 3, ***P < 0.005 statistical difference from the wild-type control. (E) Confocal imaging of WT, *ThrRS*/*NEO* and *ThrRS/NEO[pThrRS*^*+*^*]* parasites.

As the heterozygous parasites (*ThrRS*/*NEO*) showed delayed growth phenotype, there may be a likelihood of cell cycle defect, which could have reduced the growth rate of parasites. The cell cycle stages of the WT (Fig 5A), heterozygous parasites (*ThrRS*/*NEO*) (Fig 5B) and rescue mutant parasites (*ThrRS/NEO[pThrRS*^*+*^*]*) (Fig 5C) were analysed using FACS Calibur system. The heterozygous parasites showed an increased G0/G1 population of cells (60.13%) compared to the WT (51.43%) and rescue mutants (Fig 5D). This indicated G0/G1 arrest in the cell cycle of heterozygous parasites. Therefore, the G0/G1 arrest could have caused the delayed growth phenotype of heterozygous parasites (*ThrRS*/*NEO*).

The morphology of heterozygous parasites was additionally analyzed by confocal microscopy following staining with DAPI. Dramatic morphological abnormalities were observed for the heterozygous parasites (Fig 5E). Phenotypically, a large percentage (~ 64%) of heterozygous parasites were oval or shrivelled with less motility. The rescue mutants showed normal phenotype as that of WT cells.

### Inhibition studies with borrelidin

**Fig 6:**
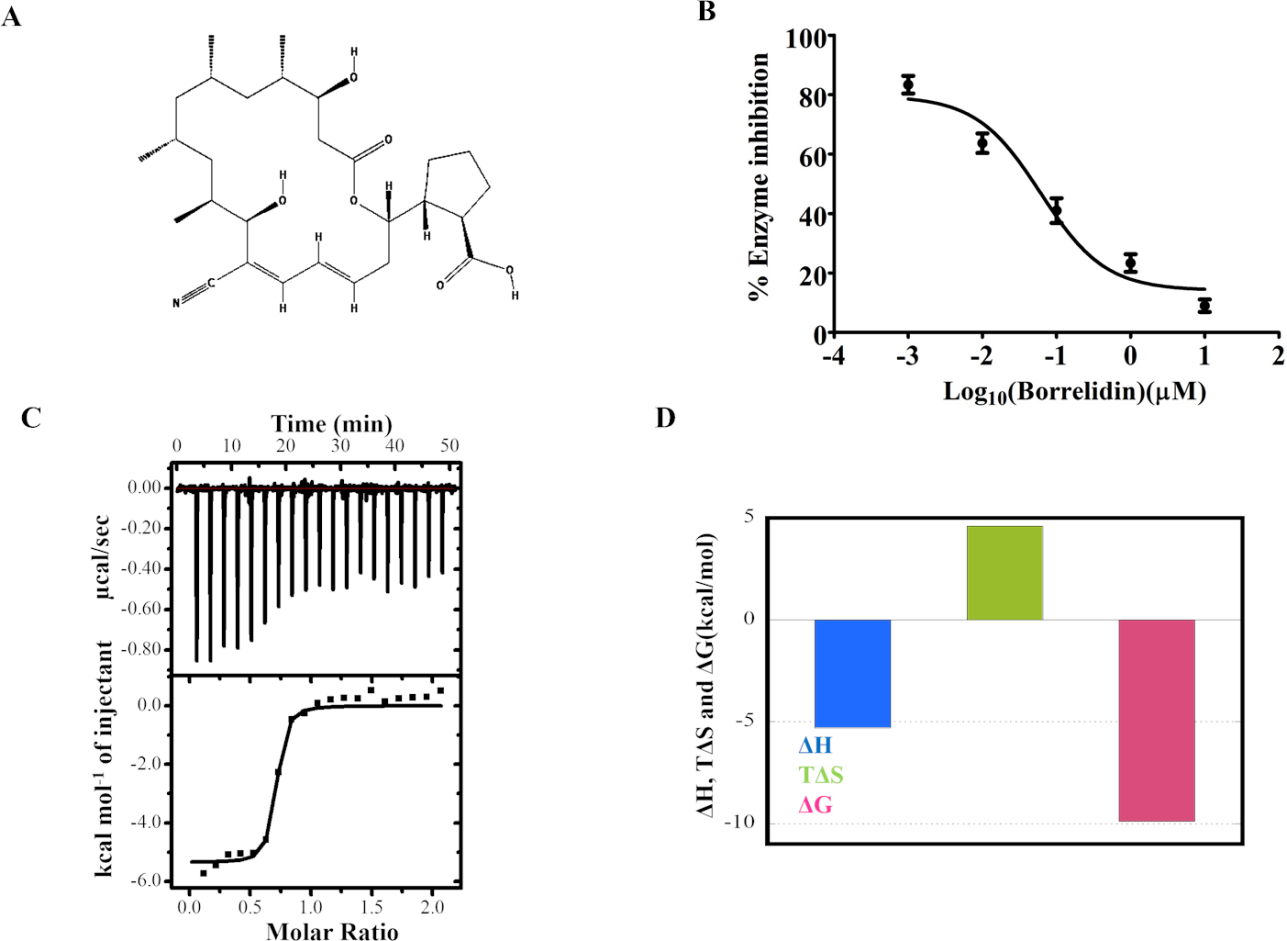
Effect of borrelidin on the r*Ld*ThrRS enzyme. (A) 2D structure of borrelidin (B) Dose-response inhibition of the aminoacylation activity of r*Ld*ThrRS in the presence of known ThrRS inhibitor, borrelidin. Inhibitor concentrations are plotted in the log scale on X-axis. The experiment was performed with 0.001–10 μM borrelidin. (C) Top Panel: ITC profile for binding of *rLd*ThrRS to borrelidin indicating the sequential injection of the drug into *rLd*ThrRS after correction of the heat of dilution of the drug. Bottom panel: Plot of integrated heat data fitted to a one-site model at 25°C. (D) Bar diagram is describing the variation of the magnitude of thermodynamic parameters of binding of borrelidin to *rLd*ThrRS.

Borrelidin is a nitrile-containing polyketide (Fig 6A) [33], first isolated from *Streptomyces* [34]. Borrelidin is a non-competitive and selective inhibitor of some of the bacterial and eukaryotic ThrRS [8]. Borrelidin has been reported to inhibit the ThrRS of *P*. *falciparum* [35], *T*. *brucei* [32] and exhibited antiplasmodial and antitrypanosomal activity. It has also been shown that this compound successfully cured mice with rodent malaria infection [36]. The inhibitory effect of borrelidin on r*Ld*ThrRS was determined. The aminoacylation activity of r*Ld*ThrRS was performed in the presence of increasing concentration of borrelidin (Fig 6B). Borrelidin inhibited the enzymatic activity of r*Ld*ThrRS with an IC_50_ (50% inhibitory concentration) of ~ 60 nM (Fig 6B). Prima facie, IC_50_ (60 nM) of the inhibitor using 400 nM of r*Ld*ThrRS appears to be discordant. This could be due to the partially active state of the r*Ld*ThrRS. It is, however, possible that limitation of the enzyme assay could have been the underline cause. In case of *T*. *brucei*, the IC_50_ for borrelidin with recombinant ThrRS under similar experimental conditions was also reported to be 66 nM [32]. Further, to determine the binding affinity of borrelidin for *Ld*ThrRS, we performed ITC (Isothermal titration calorimetry) experiments at 25°C (Fig 6C and 6D). The ITC thermogram (Fig 6C) showed the sequential injections of borrelidin into r*Ld*ThrRS resulting in exothermic heat. Borrelidin displayed strong binding affinity (K_D_ = 40 nM) to r*Ld*ThrRS with stoichiometry (N = 0.7), indicating approximately one molecule of borrelidin was bound to r*Ld*ThrRS. The reaction was driven enthalpically (ΔH = -5.3 kcal/mol) as well as entropically (TΔS = 4.6 kcal/mol) (Fig 6D). The positive entropic terms suggest the release of bound water molecules from the protein-drug interface upon complex formation. The presence of hydrogen bonds and van der Waal forces could account for the favourable ΔH value. Together, our enzyme inhibition and ITC data demonstrate strong binding affinity of borrelidin for *Ld*ThrRS.

**Fig 7:**
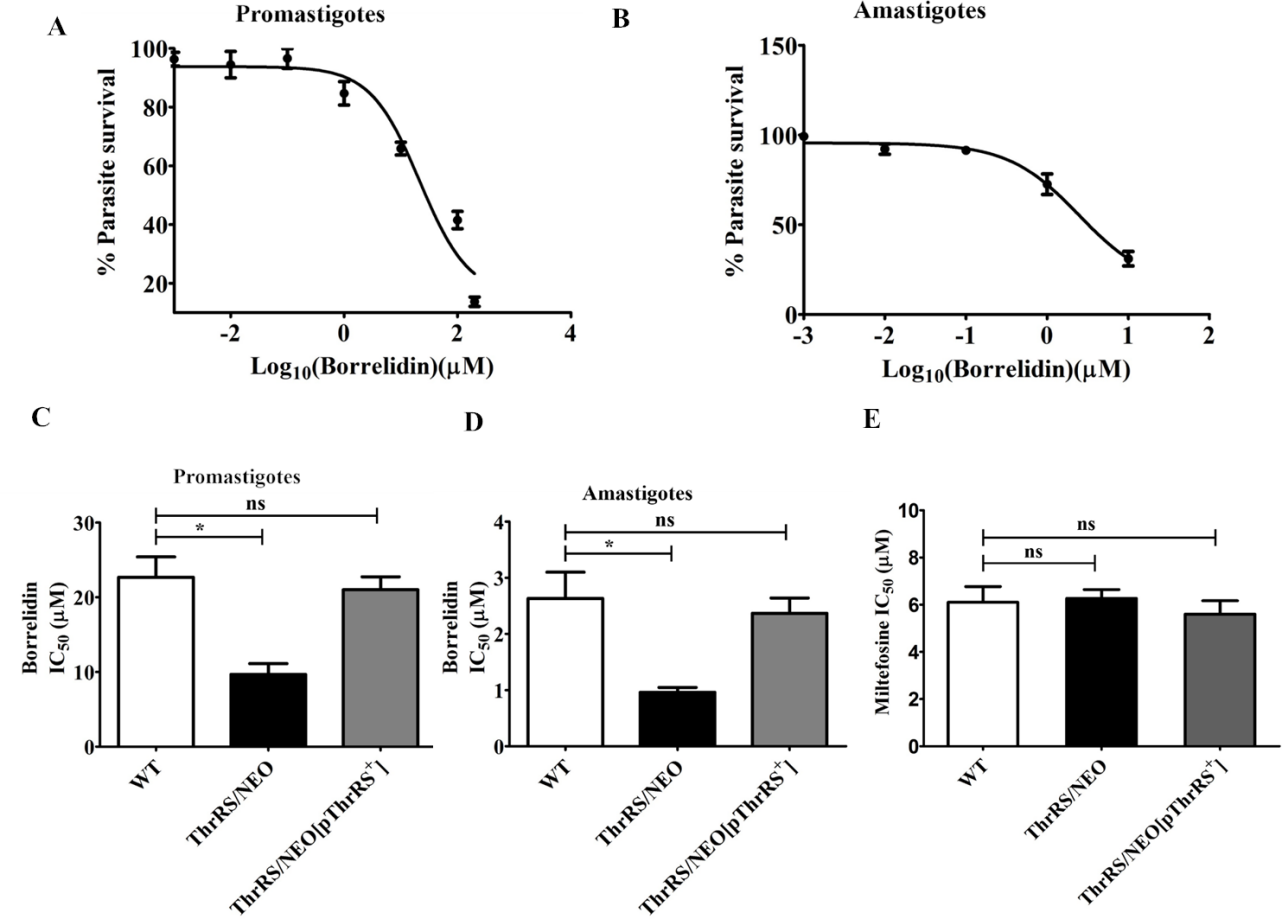
Effect of borrelidin on the growth of WT, *ThrRS/NEO* and *ThrRS/NEO[pThrRS*^*+*^*]* parasites. (A) and (B) Inhibition profile of borrelidin for the promastigote (A) and intracellular amastigote (B) growth of WT parasites. Percentage parasite survival was plotted against different concentrations of borrelidin. (C) and (D) The leishmanicidal effect of borrelidin was checked on promastigotes (C) and amastigotes (D) of WT, *ThrRS/NEO* and *ThrRS/NEO[pThrRS*^*+*^*]* parasites. The mean IC_50_ values were calculated for borrelidin and plotted as bar graphs. (E) The effect of miltefosine on WT, *ThrRS/NEO* and *ThrRS/NEO[pThrRS*^*+*^*]* promastigotes. The bar graphs represent mean ± SD with *n* = 3. * *p* < 0.05.

In order to test the effectiveness of borrelidin on *L*. *donovani*, promastigotes were grown in increasing concentration of borrelidin. The IC_50_ of borrelidin for promastigotes of WT was 21 μM (Fig 7A). The effect of borrelidin was also seen on the proliferation of intracellular amastigotes. The IC_50_ of borrelidin for amastigotes was 2.5 μM (Fig 7B). We further found borrelidin was highly cytotoxic to the host cells (CC_50_: 0.87 μM). It is quite likely that the IC_50_: 2.5 μM obtained in case of amastigotes could be a reflection of the cytotoxicity against the host cells.

To further confirm the anti-parasitic activity of borrelidin is due to inhibition of *Ld*ThrRS, we evaluated the effect of borrelidin on the growth of heterozygous (*ThrRS/NEO*) and rescue mutant (*ThrRS/NEO[pThrRS*^*+*^*]*) promastigotes (Fig 7C) and amastigotes stages of the parasites (Fig 7D). In both the stages, the IC_50_ value of borrelidin was significantly reduced in the case of heterozygous parasites (*ThrRS/NEO*) as compared to the WT cells. The IC_50_ value of borrelidin in case of rescue mutants (*ThrRS/NEO[pThrRS*^*+*^*]*) was comparable to that of WT cells. The reduced expression of ThrRS in heterozygous parasites could have resulted in increased susceptibility of these parasites. We also checked the effect of miltefosine on WT, heterozygous (*ThrRS/NEO*) and rescue mutant (*ThrRS/NEO[pThrRS*^*+*^*]*) promastigotes. We observed that IC_50_ of miltefosine was ~ 7.5 μM in all the three strains (Fig 7E). This further confirms the specificity of borrelidin for ThrRS.

### Structural modelling and comparison

**Fig 8:**
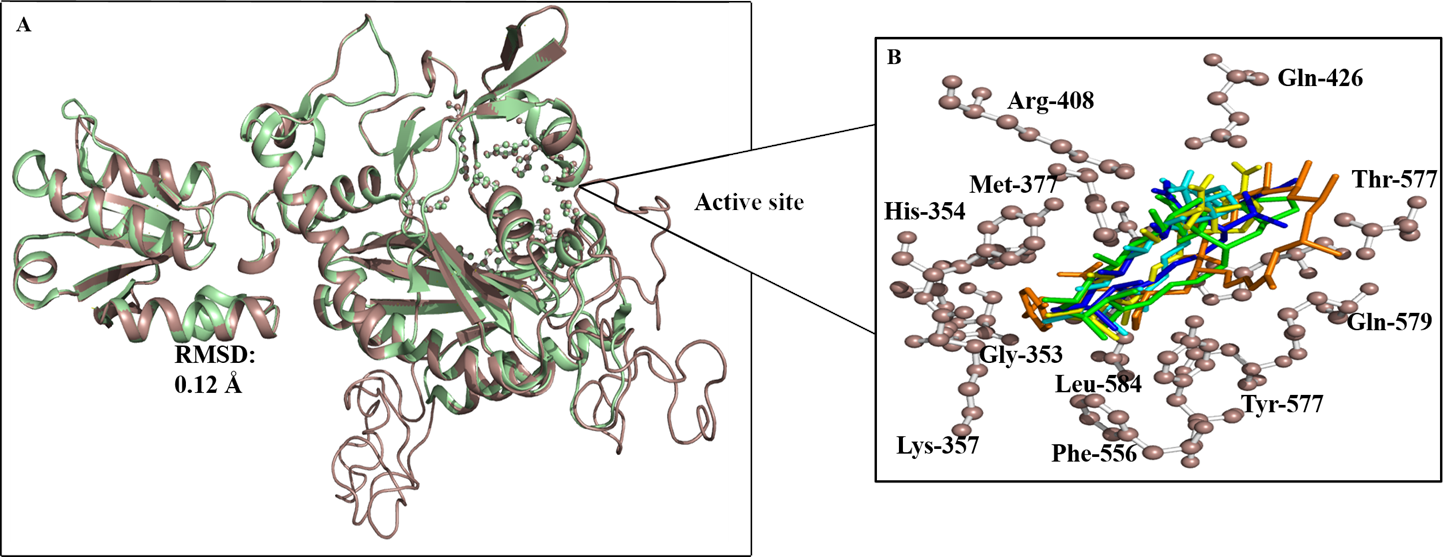
Structural modelling of *Ld*ThrRS. (A) Structural superposition of *Ld*ThrRS model with human ThrRS with an RMSD of 0.21Å. The *Ld*ThrRS protein is shown in brown color, and human ThrRS is displayed as green. (B) Active site residues are labelled as sticks.

The sequence of *Ld*ThrRS (UniProt ID: E9BS18) has shown the highest similarity against the crystal structure of human ThrRS (PDB: 4TTV), showing an identity of 60.4% and the maximum alignment score of 457 with 63% coverage. The three-dimensional structure of the *Ld*ThrRS was constructed using MODELLER8V2 [27,28] as shown in Fig 8A, covering residues from 289 to 788. The N-terminal 288 amino acids could not be modelled due to less identity. The structure was energy minimized with the CHARMM force field in order to remove poor contacts. The *Ld*ThrRS active-site residues were conserved in human ThrRS structure. The comparison of the 3D structure of *Ld*ThrRS and human ThrRS shows high similarity with an RMSD of 0.21Å (Fig 8A).

The active site residues exhibit high structural similarity between *Ld*ThrRS and human ThrRS. Identical active residues (Ser^352^, Gly^353^, His^354^, Tyr^357^, Met^377^, Cys^379^, Arg^408^, Gln^426^, Phe^556^, Tyr^557^, Thr^577^, Gln^579^, Asp^581^, Leu^584^, His^660^ except for Lys^356^ in place of His^391^) were involved in the active site cavity shown in Fig 8B.

## Discussion

Aminoacyl tRNA synthetases (aaRSs) are ubiquitous enzymes that covalently attach specific tRNAs with their cognate amino acids and ensure fidelity in the mRNA translation during protein synthesis. The aaRSs have been validated as a target for anti-microbial compounds with high druggability score [32]. It has been demonstrated in *T*. *brucei*; the gene knockdown studies of *ThrRS* by RNAi mechanism showed severe growth and phenotypic changes in the parasite, thus suggesting its strong potential to be successfully targeted for drug development [32].

We, for the first time, report the molecular characterization of ThrRS from *L*. *donovani* (*Ld*ThrRS). Kinetic analysis of recombinant *Ld*ThrRS revealed that the enzyme displayed catalytic efficiency similar to that reported in case of *T*. *brucei* [32]. In order to evaluate the potential of ThrRS as targets for drug discovery in *L*. *donovani*, we assessed the essentiality of *ThrRS* gene in *L*. *donovani* by making deletion mutants. We could replace only one allele of *ThrRS* gene with antibiotic selection gene, however our attempts to make null mutants of *ThrRS* gene failed. Heterozygous parasites (*ThrRS/NEO*) showed impaired growth and exhibited attenuated virulence. Cell cycle study of the heterozygous parasites showed a G0/G1 block which could be a likely reason for the growth defects detected in the mutant lines. Heterozygous mutants displayed reduced aminoacylation activity. Our study clearly establishes that *Ld*ThrRS is functionally essential for cell survival in *L*. *donovani*, and our data provides genetic validation for ThrRS as a potential drug target in this parasite.

While human possesses three copies of ThrRS, *Leishmania* genome encodes a single copy of ThrRS thus indicating that possibly a dual-purpose ThrRS is encoded by *L*. *donovani* which works both in the cytosol as well as in the mitochondria [16]. Therefore, this may provide a favourable opportunity to disrupt protein synthesis in two compartments simultaneously. Bioinformatic analysis has predicted to the cytosolic localization of *Ld*ThrRS. Immunofluorescence analysis of promastigotes indicates cytosolic and possibly mitochondrial localization of ThrRS.

To provide chemical substantiation of *Ld*ThrRS as a drug target, we checked the efficacy of reported ThrRS inhibitor borrelidin [8]. Our data shows that borrelidin potently inhibits r*Ld*ThrRS activity and parasite growth. Through ITC we also showed strong binding affinity of borrelidin to *Ld*ThrRS (K_D_: 0.04 μM). The specificity of the inhibitor was further confirmed by using *Ld*ThrRS heterozygous parasites. This result is consistent with the previous finding where the putative borrelidin binding site was found to be conserved in *L*. *donovani* and bacterial ThrRS (S1 Fig) [37].

In regard to the selectivity, borrelidin binding site of *L*. *donovani* ThrRS is highly conserved with the human enzyme (S1 Fig). Thus raising the concerns of mammalian toxicity. In *E*. *coli*, it has been shown that the ATP/threonine binding site has more structural differences than the borrelidin binding site and could be exploited to identify analogues of ATP and threonine [38] (S1 Fig). X-ray structure of *E*. *coli* ThrRS revealed that the replacement of Leu361 with Cys361 for the bacterial enzyme allowed the inhibitors to bind more stably with *E*.*coli* ThrRS, which was however different in case of human ThrRS. However, in case of *L*. *donovani* the putative ATP/threonine binding sites of ThrRS enzyme has a Leu residue at position 361 (*Ld*ThrRS Leu405) which is same as that of the human enzyme. Thus, ATP/threonine binding sites cannot be exploited in case of *L*. *donovani*. Instead, the differences seen near the ATP/threonine binding site in case of *Ld*ThrRS could be exploited for the identification of selective inhibitors (S1 Fig). Thus we conclude that the borrelidin binding site is highly conserved between the *L*. *donovani* and human ThrRS, suggesting that it may be difficult to target this site selectively. However, structural data is not available to entirely draw the borrelidin binding site. Also, certain analogues of borrelidin have been verified for its selectivity in case of *Plasmodium* [39].

The establishment of new drug targets in *Leishmania* through genetic and chemical validation may help in advancing the development of drugs against Leishmaniasis. In conclusion, we have validated *Ld*ThrRS as an essential enzyme and likely to bind with small drug-like molecules with high potency, making it a candidate for future drug discovery programme.

## Supporting information

S1 Fig Sequence analysis. ThrRS sequences were aligned using CLUSTAL OMEGA. Gene IDs are: Af, *A*. *fulgidus* (aful|O29703), Mj, *M*. *jannaschii* (mjann|Q58597), Ec, *E*. *coli* (ecol|P0A8M3),Tb, *T*. *brucei* (tbru|Tb927.5.1090), Li, *L*. *infantum* (lin|LINJ_35_1420), Ld, *L*. *donovani* (ldon|LDBPK_351420.1), Sc, *S*. *cerevisiae* (scer|SYTC P04801), Hs, *H*. *sapiens* (hsap|SYTC P26639). HXXXH motif is shown in green color. Conserved residues are highlighted in blue. Residues shown to be essential for borrelidin binding are highlighted in yellow [37]. The critical differences between the human and *E*. *coli* enzyme are highlighted in red that leads to species selective inhibition of the *E*. *coli* enzyme by the 11d inhibitor. Amino acids in pink are those that vary in *L*. *donovani* which is present close to the 11d binding site [38]. The four domains (TGS, putative editing (tRNA _SAD), CORE and ABD domains) of *Ld*ThrRS has been marked.
(DOCX)

S2 Fig Domain architectures of ThrRS from *E*. *coli*, *L*. *donovani*, *H*. *sapiens* and *S*. *cerevisiae*. The catalytic domain (ThrRS CORE) and anticodon binding domain (ThrRS ABD) are indicated. The associated secondary domain (tRNA_SAD) is shown in blue. The TGS domain named after the threonyl tRNA synthetase, GTPase, and SpoT protein where it occurs, has been indicated.
(DOCX)

S3 Fig Phylogenetic analysis of ThrRS homologs from kinetoplastids, mammals, apicomplexans, plants, insects, fungi, bacterial and archaeal species. The neighbour joining tree was constructed by using MUSCLE program and Fig tree software. For analysis we used following accession numbers corresponding to the species as listed from top to bottom in figure. Insecta [bdor|A0A034VKS3; mdom|T1PGK8; dmel|Q9VKB0; dbus|A0A0M4EPR8; adar|ETN67379.1; aalb|XP_019535880.1], Mammalia [mmul|XP_014995286.1; hsap|SYTC P26639; ggor|A0A212ZBR6; easi| XP_014686707.1; ptig|XP_007097493.1; oari|W5PR81; btau|Q3ZBV8; rnor|XP_006232130.1; mmus|Q9DOR2], Kinetoplastida [tbru|Tb927.5.1090; tcru|EKG01491.1; lbra|LBRM_34_1330; lmex|LMXM_34_1410; lmaj|LMJF_35_1410; lin|LINJ_35_1420; ldon|LDBPK_351420.1], Fungi [ncra|V5IKE8; scer|SYTC P04801; calb|C4YQR0], Plantae [osat|Q8LPC9; zmay|A0A1D6KSQ5; atha|Q8GZ45; bnap|A0A078G5H0; brap|M4F687], Apicomplexa [tgon|XP_002371749.1; pfal|XP_001347941.1; prei|XP_012763663.2; pyoe|XP_727721.2; pova|SBT77141.1; pviv|SCO72747.1], Bacteria [ecol|P0A8M3; mtub|P9WFT5; tthe|P56881; aaeo|O67583], Archaea [mjann|Q58597; aful|O29703; tthi|WP_055428953.1; paby|WP_010868458.1; phor|WP_010884794.1]
(DOCX)
