# Supplementary material for: Genetic manipulation of Leishmania donovani threonyl tRNA synthetase facilitates its exploration as a potential therapeutic target
Source: PLoS Negl Trop Dis. 2018 Jun 13;12(6):e0006575. doi: 10.1371/journal.pntd.0006575 (PMC6025875; doi:10.1371/journal.pntd.0006575)
Supplement: S1 Fig — ThrRS sequences were aligned using CLUSTAL OMEGA. Gene IDs are: Af, A. fulgidus (aful|O29703), Mj, M. jannaschii (mjann|Q58597), Ec, E. coli (ecol|P0A8M3),Tb, T. brucei (tbru|Tb927.5.1090), Li, L. infantum (lin|LINJ_35_1420), Ld, L. donovani (ldon|LDBPK_351420.1), Sc, S. cerevisiae (scer|SYTC P04801), Hs, H. sapiens (hsap|SYTC P26639). HXXXH motif is shown in green color. Conserved residues are highlighted in blue. Residues shown to be essential for borrelidin binding are highlighted in yellow [37]. The critical differences between the human and E. coli enzyme are highlighted in red that leads to species selective inhibition of the E. coli enzyme by the 11d inhibitor. Amino acids in pink are those that vary in L. donovani which is present close to the 11d binding site [38]. The four domains (TGS, putative editing (tRNA _SAD), CORE and ABD domains) of LdThrRS has been marked. (DOCX) [file pntd.0006575.s001.docx]

**S1 Fig.** **Sequence analysis.** ThrRS sequences were aligned using CLUSTAL OMEGA. Gene IDs are: Af, *A. fulgidus* (aful|O29703), Mj, *M. jannaschii* (mjann|Q58597), Ec, *E. coli* (ecol|P0A8M3),Tb, *T. brucei* (tbru|Tb927.5.1090), Li, *L. infantum* (lin|LINJ_35_1420), Ld, *L. donovani* (ldon|LDBPK_351420.1), Sc, *S. cerevisiae* (scer|SYTC P04801), Hs, *H. sapeiens* (hsap|SYTC P26639). HXXXH motif is shown in green color. Conserved residues are highlighted in blue. Residues shown to be important for borrelidin binding are highlighted in yellow ([1](#_ENREF_1)). The key differences between the human and *E. coli* enzyme are highlighted in red that lead to species selective inhibition of the *E. coli* enzyme by 11d inhibitor ([2](#_ENREF_2)). Amino acids in pink are those that vary in *L. donovani* which is present close to 11d binding site. The four domains (TGS, putative editing (tRNA _SAD), CORE and ABD domains) of *Ld*ThrRS has been marked.

AfThRS ------------------------------------------------------------

MjThRS ------------------------------------------------------------

EcThRS ------------------------------------------------------------

TbThRS -------------------------MATAVVGKK---KGGATVDLKQLQEPDFWKTRNAI

LiThRS ----------------------------MSGKKK---AAEATVDLATLAEPGFWRQRVEI

LdThrRS ----------------------------MSGKKK---AAEATVDLATLAEPGFWRQRVEI

ScThRSc ----MSASEAG-VTEQVKKLSVKDSSNDAVKPNKKENKKSKQQSLYLDPEPTFIEERIEM

HsThRSc MFEEKASSPSGKMGGEEKPIGAGEEKQKEGGKKKNKEGSGDGGRAELNPWPEYIYTRLEM

**TGS Domain**

AfThRS ----------------------MKLLLIHADYMEYEVKKKTK------------------

MjThRS ----------------------MKMLLIHSDYLEFEAKEKTK------------------

EcThRS ---------------------MPVITLPDGSQRHY-DHAVSPMDVALDIGPGLAKACIAG

TbThRS FDELYAAQQEK----YKSMQSPISITLNDGKQLPATSWLTTPIDIAKKLSNSLAERVVAA

LiThRS FEQLWQQQQNR----YESMKAPIKVSLPDGKVMDAESWVTCPLDIAKRLSNSLPDKVIVA

LdThrRS FEQLWQQQQNR----YESMKAPIKVSLPDGKVMDAESWVTCPLDIAKRLSNSLPDKVIVA

ScThRSc FDRLQKEYNDKV---ASMPRVPLKIVLKDGAVKEATSWETTPMDIAKGISKSLADRLCIS

HsThRSc YNILKAEHDSILAEKAEKDSKPIKVTLPDGKQVDAESWKTTPYQIACGISQGLADNTVIA

**Putative editing Domain**

**TGS Domain**

AfThRS --------LAEPFDGKGERVEEVLVAFTSVEKGDDENVVRK-----AAEAIREVAE--KV

MjThRS --------IAEETENLKGKLDECLACFIAVEREDENNPEGTAIG--AVEEIEKVAN--QL

EcThRS RVNGELVDACDLIEND------AQLSIITAKDEEGLEIIRHSCAHLLGHAIKQLWPHTKM

TbThRS RVNEEIWDLTRPFEGD------ATLELLDWDDADARHVFWHSSSHVLGYALERVFQ-TRL

LiThRS RVNEALWDLTRPFEAD------CQLELLDWDEKDAREVFWHSSSHVLGYALERIFD-TKL

LdThrRS RVNEALWDLTRPFEAD------CQLELLDWDDKDAREVFWHSSSHVLGYALERIFD-TKL

ScThRSc KVNGQLWDLDRPFEGEA--NEEIKLELLDFESDEGKKVFWHSSAHVLGESCECHLG-AHI

HsThRSc KVNNVVWDLDRPLEED------CTLELLKFEDEEAQAVYWHSSAHIMGEAMERVYG-GCL

: : . : . :

AfThRS ----NAERI-MIY--------------PYAH-LSSNLADAETAVKLLKQLEAELS--DFE

**Putative editing Domain**

MjThRS ----KVNNI-VVY--------------PYAH-LSSDLSSPETAVKVLKDIESILKERGYN

EcThRS AIGPVIDNG-FYYDVDLD-----------RTLTQEDVEALEKRM-------HELAEKNYD

TbThRS SVGPALEEGGFFYEGE-T----------NRPVTEADYTTIETAM-------QELVKMKVP

LiThRS SVGPALEEGGFFYEGL-T----------NRPVSESDYKAIESAM-------QELVKQKMP

LdThrRS SVGPALEEGGFFYEGL-T----------NRPVSESDYKAIESAM-------QELVKQKMP

ScThRSc CLGPPTDDG-FFYEMAVRDSMKDISESPERTVSQADFPGLEGVA-------KNVIKQKQK

HsThRSc CYGPPIENG-FYYDMYLEE----------GGVSSNDFSSLEALC-------KKIIKEKQA

**Putative editing Domain**

**tRNA_SAD**

: . * . : * :

AfThRS VHRSPFGWYKA---------------------------------FRISCKGHPLSELSRE

MjThRS VLRAPFGWYKA---------------------------------FKISCKGHPLSELSRK

EcThRS VIKKKVSWHEARETFANRGESYKVSILDENIAHDDKPGLYFHEEYVDMCRGPHVPNMRFC

TbThRS FQRLTVKKEDALRLF--QYTEFKSKILASKVPENGTCTVYRCGNLIDPCRGPHLPDTGRV

LiThRS YQRLEVSKEDALRLF--GYTEFKSKILASKVPDGGSCTVYRCGMLIDPCRGPHLPDTGRV

LdThrRS YQRLEVSKEDALRLF--GYTEFKSKILASKVPDGGSCTVYRCGMLIDPCRGPHLPDTGRV

ScThRSc FERLVMSKEDLLKMF--HYSKYKTYLVQTKVPDGGATTVYRCGKLIDLCVGPHIPHTGRI

HsThRSc FERLEVKKETLLAMF--KYNKFKCRILNEKVN-TPTTTVYRCGPLIDLCRGPHVRHTGKI

: . * * : .

**Putative editing Domain**

**tRNA_SAD**

AfThRS IGGEAEAEVTQALKDEEEKVVSYWYILTPEKEL-VEVEKFD--FTGYERLRKFVNYEIA-

MjThRS IVAKEEKK--------EEGEESKFYLLNPETEEIIELNENNINIIKDEELLALAKHELGI

EcThRS HHFKLMKT-----------AGAYW-RGDSNNKML--QRIYGTAWADKKALNAYL------

TbThRS KSFSLTKN-----------SSSYF-EGKAENAVL--QRVYGISFPKQTMLTEWK------

LiThRS KAFAVTKN-----------SSSYL-EGKAENEVL--QRVYGISFPKNPMLTEWK------

LdThrRS KAFAVTKN-----------SSSYF-EGKAENEVL--QRVYGISFPKNPMLTEWK------

ScThRSc KAFKLLKN-----------SSCYF-LGDATNDSL--QRVYGISFPDKKLMDAHL------

HsThRSc KALKIHKN-----------SSTYW-EGKADMETL--QRIYGISFPDPKMLKEWE------

. . :

AfThRS KRRAVDVTPPHVEYMRRLELADYEPASDSGHIRYYPKGRLVKTLLEQFITRKCIDYGAME

MjThRS REHKEHDEPPHVKFIKEKDICSYEEASDPGHFRWYPKGKLMRDLLADYVYNLVVNMGAMP

EcThRS QRLEEAAKRDHRKIGKQLDLYHMQEE-APGMVFWHNDGWTIFRELEVFVRSKLKEYQYQE

TbThRS KLQEEAARRDHRTIGRHQQLFHFHEA-SPGNAFWLPHGARIYNTLIEFQRKQYRRRGFEE

LiThRS TIQEEAAKRDHRVIGRQQNLFNFHEV-SPGSAFWLPHGARIYNTLVEFLRKKYRRCGFQE

LdThrRS TIQEEAAKRDHRVIGRQQNLFNFHEV-SPGSAFWLPHGTRIYNTLVEFLRKKYRRCGFQE

ScThRSc KFLAEASMRDHRKIGKEQELFLFNEM-SPGSCFWLPHGTRIYNTLVDLLRTEYRKRGYEE

HsThRSc KFQEEAKNRDHRKIGRDQELYFFHEL-SPGSCFFLPKGAYIYNALIEFIRSEYRKRGFQE

* : :: . * : .* : *

**ThrRS CORE**

AfThRS VETPIMYDRNHPTLRRYLERFPARQYIIKGDKREFFLRFAACFGQFLMLSNSTITYRNLP

MjThRS VETPIMYDLGNPAIREHADKFGERQYRFRQGNKELMLRFAACFGQFMMKKDMYLLPRYLP

EcThRS VKGPFMMDRVLWEKTGHWDNYKDAMFTTSSENREYCIKPMNCPGHVQIFNQGLKSYRDLP

TbThRS VVSPNMFSSRLWMVSGHWDKYADNMFLINVEKEDHGMKPMNCPGHCLMFAMQPHSYRELP

LiThRS VISPNMYSSKLWMVSGHWEKYADNMFCTKCEKEDFGLKPMNCPGHCIMFASQPHSYKELP

LdThrRS VISPNMYSSKLWMVSGHWEKYADNMFCTKCEKEDFGLKPMNCPGHCIMFASQPHSYKELP

ScThRSc VITPNMYNSKLWETSGHWANYKENMFTFEVEKETFGLKPMNCPGHCLMFKSRERSYRELP

HsThRSc VVTPNIFNSRLWMTSGHWQHYSENMFSFEVEKELFALKPMNCPGHCLMFDHRPRSWRELP

* * : . : .: : :. :: * *: : : **

AfThRS LKIYELTRYSFRKEQRGELVGLRRLRAFTMPDMHTVAKDMEQAKEEFFNQYRLSVEVLRE

**ThrRS CORE**

MjThRS LKLYELSTYSFRYEQRGELVGLKRLRCFTMPDMHTVCLNLEQAMEEFEKQFWECLKTGDD

EcThRS LRMAEFGS-CHRNEPSGSLHGLMRVRGFTQDDAHIFCT-EEQIRDEVNGCIRLVYDMYST

TbThRS IRYADFGV-LHRNELSGALTGLTRVRRFQQDDAHIFCR-MDQVKDEIHSALIFLKDVYDV

LiThRS IRYADFGV-LHRNELSGALTGLTRVRRFQQDDAHIFCR-MDQITDEMEGQMQFLSDVYGV

LdThrRS IRYADFGV-LHRNELSGALTGLTRVRRFQQDDAHIFCR-MDQITDEMEGQMQFLSDVYGV

ScThRSc WRVADFGV-IHRNEFSGALSGLTRVRRFQQDDAHIFCT-HDQIESEIENIFNFLQYIYGV

HsThRSc LRLADFGV-LHRNELSGALTGLTRVRRFQQDDAHIFCA-MEQIEDEIKGCLDFLRTVYSV

: :: .* * * * ** *:* * * * .. :* .*.

**ThrRS CORE**

AfThRS IGLEPEDYEVAVRITK-----DFYEENREFVHSLVD-----------ILQ----------

MjThRS L---NLSYSVIFRFTK-----DFFDEHRDWFFKIAKEY-------KNKYG----------

EcThRS FGFEKIVVKLSTRPEKRIGSDEMWDRAEADLAVALEEN----------------------

TbThRS LGFKF-FLKHSTRPENKLGSDEMWDEAESYLQAALNSFCGIPDELPDPFNKGSTFVYDGR

LiThRS LGFKF-YFYHSTRPANKLGSEALWDQSESYLRTALNRFCGIPEELPDPLHPDQRFHYDGK

LdThrRS LGFKF-YFYHSTRPANKLGSDALWDQSESYLRTALNRFCGIPEELPDPLHPDQRFHYDGK

ScThRSc FGFEF-KMELSTRPEKYVGKIETWDAAESKLESALKKW----------------------

HsThRSc FGFSF-KLNLSTRPEKFLGDIEVWDQAEKQLENSLNEF----------------------

: * : :: . . .

**ThrRS CORE**

AfThRS ----------------------------------KPILIEMWDHRFFYFVLKFEFNFVDA

MjThRS ----------------------------------KDVILEILPKRKHYWVGKVDIAVIDS

EcThRS -----------------------------------NIPFEYQLGEGAFYGPKIEFTLYDC

TbThRS KESVKKLRAMLKKRGDKKNDENSPADDVWPGPTH-EDAWEENTGDGAFYGPKIDIVVEDA

LiThRS PDSVKKMKALMKKAE------RCEDPNAWKGPTNGGDGWIENAGDGAFYGPKIDIVVEDA

LdThrRS PDSVKKMKALMKKAE------RCEDPNAWKGPTNGGDGWIENAGDGAFYGPKIDIVVEDA

ScThRSc -----------------------------------GGNWEINAGDGAFYGPKIDIMISDA

HsThRSc -----------------------------------GEKWELNSGDGAFYGPKIDIQIKDA

:: *.:: . *.

**ThrRS CORE**

AfThRS LDKASALSTVQIDVENAERYGITFVDSDG-------------------------------

MjThRS LGRPIENPTVQIDVESAKRFDIKVHTNEG-------------------------------

EcThRS LDRAWQCGTVQLDFSLPSRLSASYVGEDN-------------------------------

TbThRS LRRRHQCATIQLDFNLPKRFGLKYTLPSNAAGDDGPAPKATECHKDPNAELKADKPEGKV

LiThRS LRRRHQCATIQLDFNLPSRFGLKYTLPAAEKDETTVSPTEKQKHADPALTASSPAVE---

LdThrRS LRRRHQCATIQLDFNLPSRFGLKYTLPAAEKDETTVSPTEKQKHADPALTASSPAVE---

ScThRSc LRRWHQCATIQLDFQLPNRFELEFKSKDQD------------------------------

HsThRSc IGRYHQCATIQLDFQLPIRFNLTYVSHDGD------------------------------

: : *:*:*.. *

AfThRS --------------------------KEKHPYILHCSVSGAVERVMYALLEKAKFMLDEG

MjThRS ---------------------------EIYPIILHCSPTGSIERVLCGLLEKAAIEAEKG

EcThRS --------------------------ERKVPVMIHRAILGSMERFIGILTE--------E

TbThRS EAAAGAPKSSYEKAAHELNIDQRLDANQARPVMIHRAIFGSLERCIAILCE--------H

LiThRS DCVSKPKPGTYEAAVRDLGIDLELDANQARPVMIHRAILGSLERSIAILCE--------H

LdThrRS DCVSKPKPGTYEAAVRDLGIDLELDANQARPVMIHRAILGSLERSIAILCE--------H

ScThRSc ------------------------SESYERPVMIHRAILGSVERMTAILTE--------H

HsThRSc --------------------------DKKRPVIVHRAILGSVERMIAILTE--------N

* ::* : *::** * *

**ThrRS ABD**

AfThRS MLPMLPVWLSPTQVRVIPVSERFVDAAIKIADDIARNGIRVDVDDR-NETLGKKIRDAQT

MjThRS NAPMLPVWLSPIQVRVIPVAERHYDYALKVAEKLRENNIRADFDDR-EESVSKKIRNAGK

EcThRS FAGFFPTWLAPVQVVIMNITDSQSEYVNELTQKLSNAGIRVKADLR-NEKIGFKIREHTL

TbThRS YGGDWPFWLSPRQVIVVPVSLENAAYAQTVRDTFFAAGFFADVDNG-TATLEKKIRNAEL

LiThRS FGGDWPFWLSPRQVMVVPVSASNYEYSQQVRDTMHDAGFHADVDNG-AATLDKKIRNAEK

LdThrRS FGGDWPFWLSPRQVMVVPVSASNYEYSQQVRDTMHDAGFHADVDNG-AATLDKKIRNAEK

ScThRSc FAGKWPFWLSPRQVLVVPVGVKYQGYAEDVRNKLHDAGFYADVDLT-GNTLQKKVRNGQM

HsThRSc YGGKWPFWLSPRQVMVVPVGPTCDEYAQKVRQQFHDAKFMADIDLDPGCTLNKKIRNAQL

* **:* ** :: : : : : : .. * .: *:*:

**ThrRS ABD**

AfThRS EWIPYIAVVGEKEIESGKLAVTVRAESTQKEQKRVEMSAEELAKRVRAECE---GKPFMP

MjThRS EWVPYVVVIGDEEMESDKLTVTIREKSTLKKPYKEKMTLDELIERIKKETA---NYPYRP

EcThRS RRVPYMLVCGDKEVESGKVAVRTRRGKDLGSMDVN-EVIEKLQQEIRSRSLKQLEE----

TbThRS ARYNFILVVGQAEQEGQSVNVRTRDNRRHGTKTLD-----EALQWLRELADKYDPSF---

LiThRS ARYNFILVVGQKEQDATAVNIRARGEKRLGNKSLV-----EAVQWLKELADTHNREY---

LdThrRS ARYNFILVVGQKEQDATAVNIRARGEKRLGNKSLV-----EAVQWLKELADTHNREY---

ScThRSc LKYNFIFIVGEQEMNEKSVNIRNRDVMEQQGKNAT-VSVEEVLKQLRNLKDEKRGDNVLA

HsThRSc AQYNFILVVGEKEKISGTVNIRTRDNKVHGERTIS-ET-IERLQQLKEFRSKQAEEEF--

:: : *: * : : * : : ::

AfThRS LPLPKLLSLRPSFR

MjThRS LPLPIRCSLQPKFH

EcThRS --------------

TbThRS --------------

LiThRS --------------

LdThrRS --------------

ScThRSc --------------

HsThRSc --------------

**References**

1. Ruan, B., Bovee, M. L., Sacher, M., Stathopoulos, C., Poralla, K., Francklyn, C. S., and Soll, D. (2005) A unique hydrophobic cluster near the active site contributes to differences in borrelidin inhibition among threonyl-tRNA synthetases. *The Journal of biological chemistry* **280**, 571-577

2. Teng, M., Hilgers, M. T., Cunningham, M. L., Borchardt, A., Locke, J. B., Abraham, S., Haley, G., Kwan, B. P., Hall, C., Hough, G. W., Shaw, K. J., and Finn, J. (2013) Identification of bacteria-selective threonyl-tRNA synthetase substrate inhibitors by structure-based design. *Journal of medicinal chemistry* **56**, 1748-1760
