# Supplementary material for: Genetic manipulation of Leishmania donovani threonyl tRNA synthetase facilitates its exploration as a potential therapeutic target
Source: PLoS Negl Trop Dis. 2018 Jun 13;12(6):e0006575. doi: 10.1371/journal.pntd.0006575 (PMC6025875; doi:10.1371/journal.pntd.0006575)

**S2 Fig. Domain architectures of ThrRS from *E. coli*, *L. donovani*, *H. sapiens* and *S. cerevisiae*.** The catalytic domain (ThrRS CORE) and anticodon binding domain (ThrRS ABD) are indicated. The associated secondary domain (tRNA_SAD) is shown in blue. The TGS domain named after the threonyl tRNA synthetase, GTPase, and SpoT protein where it occurs, has been indicated.


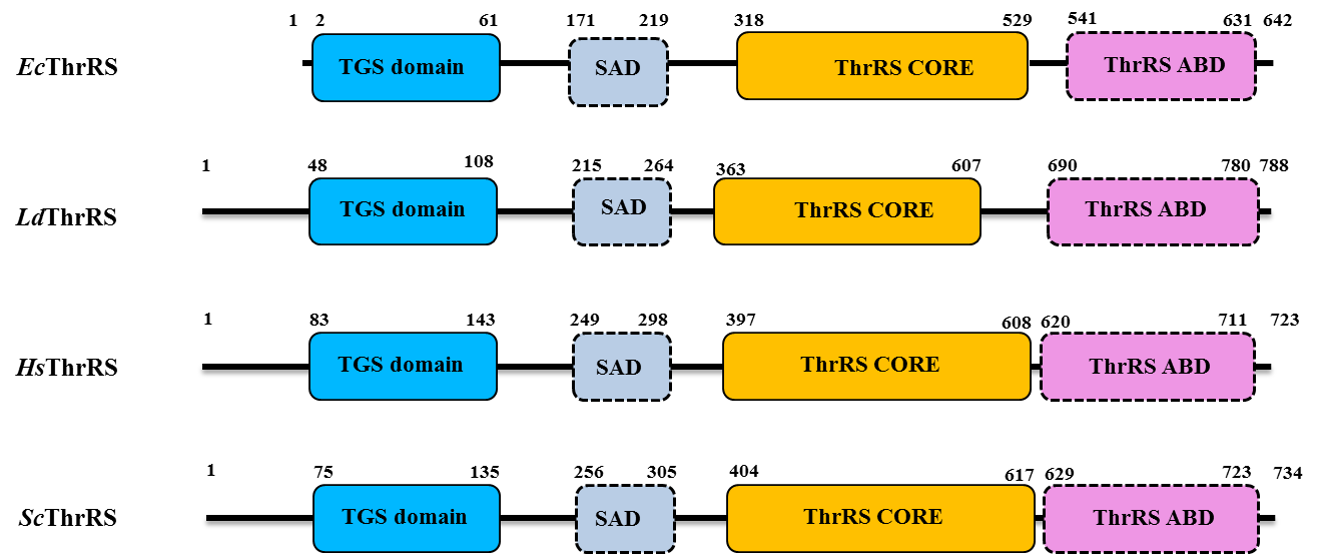

Supplement: S2 Fig — The catalytic domain (ThrRS CORE) and anticodon binding domain (ThrRS ABD) are indicated. The associated secondary domain (tRNA_SAD) is shown in blue. The TGS domain named after the threonyl tRNA synthetase, GTPase, and SpoT protein where it occurs, has been indicated. (DOCX) [file pntd.0006575.s002.docx]
