# Supplementary material for: Genetic manipulation of Leishmania donovani threonyl tRNA synthetase facilitates its exploration as a potential therapeutic target
Source: PLoS Negl Trop Dis. 2018 Jun 13;12(6):e0006575. doi: 10.1371/journal.pntd.0006575 (PMC6025875; doi:10.1371/journal.pntd.0006575)
Supplement: S3 Fig — The neighbour joining tree was constructed by using MUSCLE program and Fig tree software. For analysis we used following accession numbers corresponding to the species as listed from top to bottom in figure. Insecta [bdor|A0A034VKS3; mdom|T1PGK8; dmel|Q9VKB0; dbus|A0A0M4EPR8; adar|ETN67379.1; aalb|XP_019535880.1], Mammalia [mmul|XP_014995286.1; hsap|SYTC P26639; ggor|A0A212ZBR6; easi| XP_014686707.1; ptig|XP_007097493.1; oari|W5PR81; btau|Q3ZBV8; rnor|XP_006232130.1; mmus|Q9DOR2], Kinetoplastida [tbru|Tb927.5.1090; tcru|EKG01491.1; lbra|LBRM_34_1330; lmex|LMXM_34_1410; lmaj|LMJF_35_1410; lin|LINJ_35_1420; ldon|LDBPK_351420.1], Fungi [ncra|V5IKE8; scer|SYTC P04801; calb|C4YQR0], Plantae [osat|Q8LPC9; zmay|A0A1D6KSQ5; atha|Q8GZ45; bnap|A0A078G5H0; brap|M4F687], Apicomplexa [tgon|XP_002371749.1; pfal|XP_001347941.1; prei|XP_012763663.2; pyoe|XP_727721.2; pova|SBT77141.1; pviv|SCO72747.1], Bacteria [ecol|P0A8M3; mtub|P9WFT5; tthe|P56881; aaeo|O67583], Archaea [mjann|Q58597; aful|O29703; tthi|WP_055428953.1; paby|WP_010868458.1; phor|WP_010884794.1] (DOCX) [file pntd.0006575.s003.docx]

**S3 Fig. Phylogenetic analysis of ThrRS homologs from kinetoplastids, mammals, apicomplexans, plants, insects, fungi, bacterial and archaeal species.** The neighbour joining tree was constructed by using MUSCLE program and Fig tree software. For analysis we used following accession numbers corresponding to the species as listed from top to bottom in figure. Insecta [bdor|A0A034VKS3; mdom|T1PGK8; dmel|Q9VKB0; dbus|A0A0M4EPR8; adar|ETN67379.1; aalb|XP_019535880.1], Mammalia [mmul|XP_014995286.1; hsap|SYTC P26639; ggor|A0A212ZBR6; easi| XP_014686707.1; ptig|XP_007097493.1; oari|W5PR81; btau|Q3ZBV8; rnor|XP_006232130.1; mmus|Q9DOR2], Kinetoplastida [tbru|Tb927.5.1090; tcru|EKG01491.1; lbra|LBRM_34_1330; lmex|LMXM_34_1410; lmaj|LMJF_35_1410; lin|LINJ_35_1420; ldon|LDBPK_351420.1], Fungi [ncra|V5IKE8; scer|SYTC P04801; calb|C4YQR0], Plantae [osat|Q8LPC9; zmay|A0A1D6KSQ5; atha|Q8GZ45; bnap|A0A078G5H0; brap|M4F687], Apicomplexa [tgon|XP_002371749.1; pfal|XP_001347941.1; prei|XP_012763663.2; pyoe|XP_727721.2; pova|SBT77141.1; pviv|SCO72747.1], Bacteria [ecol|P0A8M3; mtub|P9WFT5; tthe|P56881; aaeo|O67583], Archaea [mjann|Q58597; aful|O29703; tthi|WP_055428953.1; paby|WP_010868458.1; phor|WP_010884794.1]

**
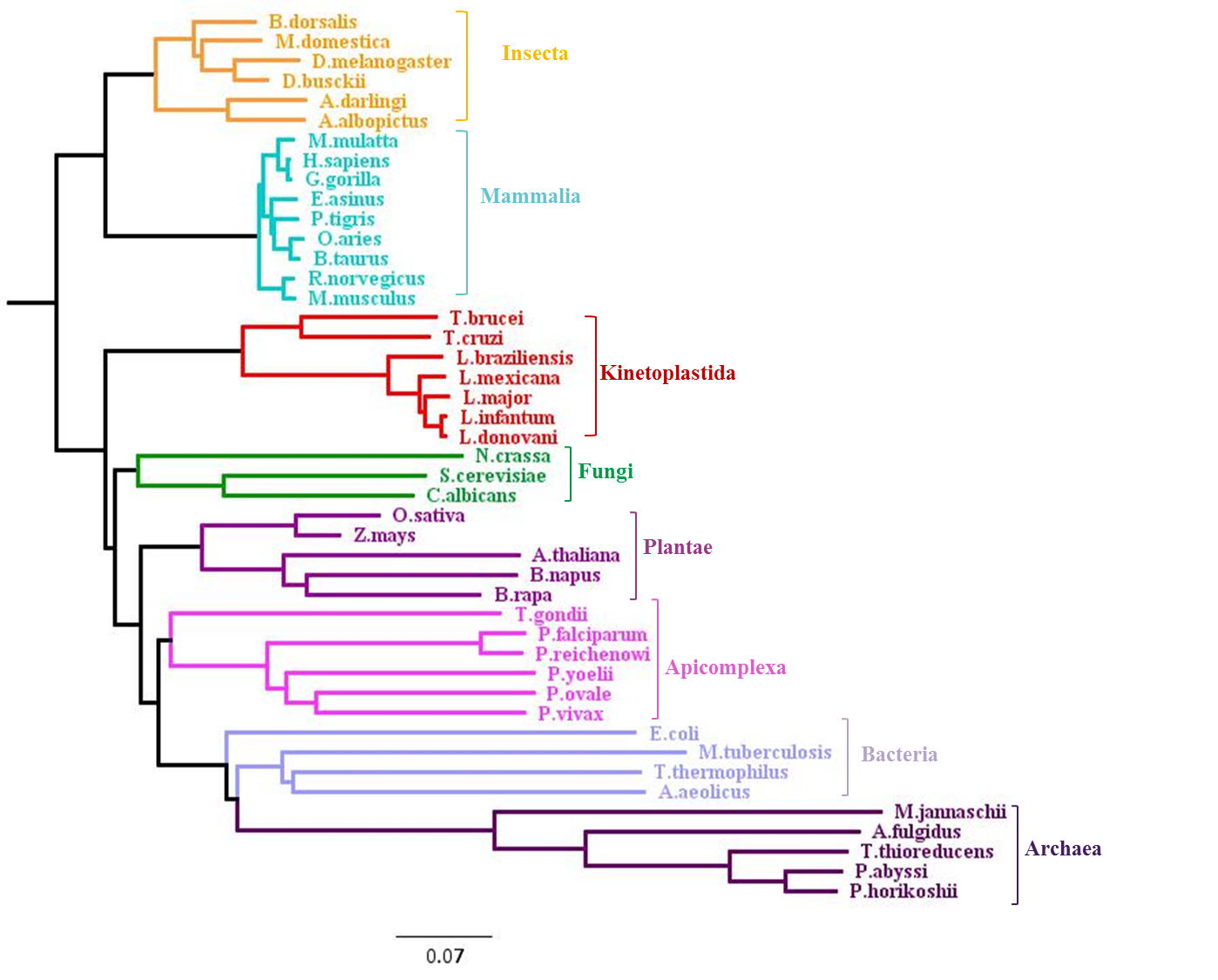
**
